# Endostatin 33 Peptide Is a Deintegrin α6β1 Agent That Exerts Antitumor Activity by Inhibiting the PI3K-Akt Signaling Pathway in Prostate Cancer

**DOI:** 10.3390/jcm12051861

**Published:** 2023-02-27

**Authors:** Yang Liu, Chang-Lin Wang, Zhong-Qi Pang, Ke Gao, Lin-Kun Shen, Wan-Hai Xu, Ming-Hua Ren

**Affiliations:** 1 Department of Urology, The First Affiliated Hospital of Harbin Medical University, Harbin 150001, China; 202101233@hrbmu.edu.cn (Y.L.); 2021020849@hrbmu.edu.cn (Z.-Q.P.); 2022020675@hrbmu.edu.cn (K.G.); 2020020828@hrbmu.edu.cn (L.-K.S.); 2 Department of Urology, The Fourth Affiliated Hospital of Harbin Medical University, Harbin 150001, China; 601740@hrbmu.edu.cn

**Keywords:** endostatin 33 polypeptide, QRD, integrin α6β1, PI3K-AKT, prostate cancer

## Abstract

Background: Prostate cancer (PCa) is the leading cause of death in men and has poor therapeutic outcomes. Methods: A novel endostatin 33 peptide was synthesized by adding a specific QRD sequence on the basis of the endostatin 30 peptide (PEP06) with antitumor activity. Then, bioinformatic analysis and subsequent experiments were performed to validate the antitumor function of this endostatin 33 peptide. Results: We found that the 33 polypeptides significantly inhibited growth, invasion and metastasis and promoted the apoptosis of PCa in vivo or vitro, which is more significant than PEP06 under the same conditions. According to 489 cases from the TCGA data portal, the α6β1 high expression group was closely associated with the poor prognosis (Gleason score, pathological N stage, etc.) of PCa and was mainly enriched in the PI3K-Akt pathway. Subsequently, we demonstrated that endostatin 33 peptide can down-regulate the PI3K-Akt pathway via the targeted inhibition of α6β1, thereby inhibiting the epithelial–mesenchymal transition and matrix metalloproteinase in C42 cell lines. Conclusion: The endostatin 33 peptide can exert antitumor effects by inhibiting the PI3K-Akt pathway, especially in tumors with a high expression of the integrin α6β1 subtype, such as prostate cancer. Therefore, our study will provide a new method and theoretical basis for the treatment of prostate cancer.

## 1. Introduction

Prostate cancer (PCa) is the most common malignant tumor in men in Western countries, with the second highest mortality rate [1]. It has been reported that the treatment effects of PCa were disappointing, especially for castration-resistant prostate cancer (CRPC), for which the success rate of combination therapy was less than 30% [2]. Therefore, it is particularly urgent and necessary to explore new treatment methods for PCa.

Endostatin is a factor that consists of 184 amino acid residues and can inhibit angiogenesis, which was first obtained from mouse vascular endothelial cells by Folkman et al. [3]. Integrin belongs to a family of 24 heterodimer cell-surface receptors, which are composed of non-covalently bound α and β subunits and play an important role in angiogenesis [4]. Integrins such as αvβ1, αvβ3, αvβ5 and αvβ6 can recognize the RGD (arginine–glycine–aspartic acid) sequence of extracellular matrix (ECM) proteins and contribute to cancer and its metastasis [5]. In endostatin, a sequence with a similar structure to the RGD sequence binds to integrin, thus exerting antitumor effects in tumor tissues [6]. However, endostatin is a large-molecular-weight protein, which is difficult to separate and purify, and has a relatively weak anti-tumor effect, limiting its application in a clinical setting [7].

Integrin αvβ3 is overexpressed in tumors and is one of the most studied integrins [8,9]. αvβ3 mediates adhesion, invasion, immune escape and neovascularization via interactions with different ligands [10]. RGD sequences are present in most of the natural ligand structures of αvβ3 and have a high affinity for αvβ3 integrin receptors, which have been a target of cancer therapy [8,11]. In order to reduce the defects of endostatin, we mutated the RGIRGAD sequence at the 25–31 position of human endostatin into the RGDRGD sequence in our previous works so that endostatin could bind to integrin αvβ3 on the tumor cell membrane via the RGD sequence, and we obtained mutated endostatin with stronger anti-tumor activity [12]. Subsequently, Yang B et al. simplified the mutagenic endostatin, retaining the first 24 amino acids in the active region of anti-angiogenesis, and added the RGDRGD sequence to the terminal, obtaining PEP06. Additionally, studies showed that PEP06 had good therapeutic effects on a variety of tumors [7,13,14]. 

Integrin α6β1 has been reported to be expressed in cancer and endothelial cells and has been described as beneficial to tumor angiogenesis, invasion and cancer progression [15]. In fact, integrin α6β1 is highly expressed in PCa tissues, especially in metastatic and androgen receptor (AR)-positive Pca [16,17,18,19], and it is not recognized by the RGD sequence. Therefore, PEP06 is not effective in treating tumors with the α6β1 integrin subtype, especially for PCa. The α6β1 integrin can bind to the QRD (glutamine-arginine-aspartic acid) sequence of the extracellular large period-specific site of CD151 to form a stable complex [20,21], suggesting that the QRD sequence is a recognition sequence of α6β1 integrin. Accordingly, we further modified PEP06 and synthesized the 33 polypeptide by adding the QRD sequence to the N-terminal of PEP06. In this study, we aimed to further investigate the antitumor activity and mechanism of 33 polypeptide in PCa. Our study will provide a new method and theoretical basis for the treatment of PCa.

## 2. Materials and Methods

### 2.1. Data Sources

In our study, 489 cases of prostate cancer were acquired from the TCGA data portal (https://tcga-data.nci.nih.gov/tcga/) (accessed on 1 February 2022), of which transcriptomics data and corresponding clinical information were included.

### 2.2. Weighted Gene Co-Expression Network Analysis (WGCNA)

In our study, WGCNA was used to search for co-expressed gene modules and explore the association between gene modules and clinical traits.

### 2.3. Functional Enrichment Analysis

In our study, the KEGG database (https://www.genome.jp/kegg/pathway.html) (accessed on 5 February 2022) was used to identify the pathways involved in the gene modules of WGCNA.

### 2.4. Protein–Protein Interaction (PPI) Network

The PPI network (https://cn.string-db.org/) (accessed on 10 February 2022) was used to evaluate the interactions and regulatory relationships between integrin α6β1, PI3K-Akt, epithelial–mesenchymal transition (EMT) and matrix metalloproteinase (MMPs).

### 2.5. Materials and Reagents

The human prostate cancer cell lines C4-2, 22RV1 and DU145 were purchased from the Institute of Biochemistry and Cell Biology, Chinese Academy of Sciences (Shanghai, China). The PEP06 and 33 polypeptide were synthesized by Ningbo Kangbei Biochemical Co., Ltd. (Ningbo, China). RPMI 1640 medium was purchased from Thermo Fischer Scientific Inc.(Waltham, MA, USA) FBS, penicillin and streptomycin were purchased from HyClone Laboratories Inc. (Logan, UT, USA). Trypsin was purchased from Invitrogen (Carlsbad, CA, USA). Matrigel basement membrane matrix was purchased from Seebio Biotechnology Co., Ltd. (Shanghai, China). Dimethyl sulfoxide (DMSO) was purchased from VWR International Co. Transwell cell inserts were purchased from Wuxi NEST Biotechnology Co., Ltd. (Wuxi, China) Rabbit anti-αv, anti-β3, anti-α6, anti-β1, anti-MMP2, anti-E-cadherin, anti-β-catenin, anti-vimentin, anti-MMP9, anti-AKT and anti-P-AKT monoclonal antibodies were purchased from Abcam Trading Co., Ltd. (Shanghai, China).

### 2.6. Cell Culture

The C4-2, 22RV1 and DU145 cells were cultured in RPMI-1640 medium supplemented with 10% fetal bovine serum and 1% penicillin/streptomycin at 37 °C and in 5% CO_2_. Cells were subcultured by the trypsinization of subconfluent cultures using 0.25% trypsin. 

### 2.7. MTT Cell Viability Assay

Prostate cancer cells (C4-2, 22RV1 and DU145) were seeded in 96-well plates (4–8 × 10^3^ cells/well) and allowed to attach overnight. The cells were incubated in a serum-free medium for 24 or 48 h and then treated with different concentrations of PEP06, 33 polypeptide, glucose solution, cisplatin or 5-fluorouracil. After culturing for 24 h, 20 μL of MTT solution (5 mg/mL) was added to each well, and incubation continued at 37 °C for 4 h. The supernatant was discarded, 150 µL DMSO was added to each well, and the plate was shaken for 10 min until the crystalline solid was completely dissolved. Then, absorbance at 490 nm was measured with a spectrophotometer.

### 2.8. Cell Adhesion Assay

Briefly, 96-well plates were coated with 2 μg of Matrigel overnight at 4 °C. Next, 20 μL of 2% BSA was added to each well for 1 h, followed by washing with PBS. Moreover, C4-2 cells were treated with PEP06 or 33 polypeptide at 100 or 200 μg/mL for 24 h. A 5% glucose solution was used as the negative control. After culturing for 24 h, 20 μL of MTT solution (5 mg/mL) was added to each well, and incubation continued at 37 °C for 4 h. The supernatant was discarded, and 150 µL DMSO was added to each well. Then, absorbance at 570 nm was measured with a spectrophotometer.

### 2.9. Transwell Assay

Migration assay: the cells were incubated in serum-free medium for 12 h and then adjusted to a density of 1 × 10^6^ cells/mL. Next, 100 μL of cell suspension containing different concentrations of PEP06 or 33 polypeptide was added to the transwell chambers, which were fit into the wells of 24-well plates. The wells of the plates also each contained 600 μL of RPMI-1640 medium supplemented with 10% FBS. After 24 h of incubation, cells on the upper membrane were removed with cotton wool, whereas cells adhering to the lower surface were fixed in methanol for 30 min and then stained with 0.1% crystal violet for 20 min. After natural air drying, the migrating cells on the lower surface of the membrane were counted under an optical microscope at 400× magnification.

Invasion assay: Matrigel was diluted with serum-free medium (1:8) on ice, and 30 μL was added to the upper chambers of the transwell. Then, the upper chambers were transferred to a cell culture incubator to allow Matrigel solidification for at least 5 h. Next, the experiment was implemented following the same procedure as that used for the migration experiment.

### 2.10. Cell Apoptosis Assay

Prostate cancer cells were seeded in six-well plates (2 × 10^4^ cells/well), allowed to attach overnight and then treated with the corresponding concentrations of PEP06 or 33 polypeptide. After culturing for 24 or 48 h, the acridine orange and ethidium bromide (AO/EB) dye mix (100 μg/mL of AO and 100 μg/mL of EB) was prepared, and 10 μL was added to each well and mixed well. After 2–3 min, the mixed solution was discarded, and the cells were observed under a fluorescence microscope.

### 2.11. Wound Healing Assay

C4-2 or 22RV1 cells were seeded onto six-well plates without peptide treatment until the cells grew into a confluent single layer. The treatment groups received corresponding concentrations of PEP06 or 33 polypeptide, whereas the negative control received 5% glucose solution. At 24 h after treatment, cells were scratched using a sterile 200 μL pipette tip. The width of the line was recorded and photographed at 0, 6, 12, 24 and 48 h after wounding.

### 2.12. SiRNA Transfections

According to research requirements, the predesigned specific siRNA sequences were used to knock out the integrin subunits αv, β3, α6 and β1. These sequences were as follows: αv-siRNA (sequence: 5′-GTAGTCAATCTCTATCAGA-3′); β3-siRNA (sequence: 5′-GTGCAATCTTGTACGTAAA-3′); α6-siRNA (sequence: 5′-CTTAGG CTGTTACATCTCTCC-3′); β1-siRNA (sequence: 5′-GGAG AACCACAGAAGTTT A-3′); and negative control siRNA (AM4635). Briefly, the day before transfection, cells were inoculated in 6-well plates with 0.5–2 × 105 cells per well. The cells were cultured in antibiotic-free medium and transfected when the cells reached 40–60% fusion degree on the second day. The medium was changed to 1.5 mL serum-free Opti-MEM half an hour before transfection. Transfection complex was prepared: tube 1:245 µL Opti-medium+5 µL lipo2000; tube 2:240 µL Opti-medium+10 µL siRNA. Tube 1 and tube 2 were thoroughly mixed and stood still at room temperature for 10 min and then dropped into the wells to be transfected. After transfection, the cells were cultured in an incubator at 37 °C and 5% CO_2_. Cells were used for experiments 24 h after transfection.

### 2.13. Western Immunoblotting

Forty micrograms of total protein, as determined using a BCA protein assay kit, was separated by SDS-PAGE on 12% gradient polyacrylamide gels. Gels were electroblotted onto nitrocellulose membranes. For immunodetection, blots were blocked with 1% blocking reagent in 0.05% Tween 20-PBS for 1 h and incubated with primary antibody overnight at 4 °C diluted in blocking buffer. The dilutions used in Western blots were anti-αv (1:5000), anti-β3 (1:1000), anti-α6 (1:2000), anti-β1 (1:2000), anti-AKT (1:1000), anti-P-AKT (1:1000), anti-E-cadherin (1:2000), anti-β-catenin (1:1000), anti-vimentin (1:1000), anti-MMP2 (1:2000), anti-MMP9 (1:5000) and β-actin (1:5000). Blots were then washed in 0.05% Tween 20-PBS and incubated with either goat anti-mouse (1:5000) or goat anti-rabbit (1:5000) peroxidase-labeled antibodies in a blocking buffer for 1 h. An enhanced chemoluminescent system was applied. Scanning densitometry was performed with scan analysis software. 

### 2.14. Xenograft Tumor Model

We established the xenograft tumor model by using male BALB/c nude mice (6–8 weeks old) under anesthetic conditions. At first, a total of 18 BALB/c mice were divided into 3 groups randomly, with 6 mice in each group. These 3 groups comprised the control group, the PEP06-treated group and the peptide 33-treated group. Then, we injected prostate cancer cell line C4-2 (1 × 106 cells in 50 μL RPMI-1640 medium) under the skin of the right armpit of each mouse to establish the xenograft tumor model. Meanwhile, PEP06 and peptide 33 were dissolved in 5% glucose saline solution to reach a concentration of 50 µg/mL, and 100 µL of the treating solution would be injected to every mouse via the tail vein every day: 5% glucose saline solution to the control group, PEP06 to the PEP06 group and peptide 33 to the peptide 33 group. In addition, we examined the size of the tumor every day. After 4 weeks of follow-up, the subcutaneous tumors of the 3 groups of mice were excised, the tumor size, weight and volume were measured, and statistical analysis was performed (*p* < 0.05 was considered statistically significant).

### 2.15. Statistical Analysis

All results are expressed as the mean ± standard deviation (SD). Student’s *t* test was used to compare differences between two groups, and one-way analysis of variance was used to compare the difference between three or more groups. Statistical analysis was performed using GraphPad Prism version 5.0. Heatmap and correlation analysis were plotted by R version 3.5.1. All experiments were independently repeated three times (n = 3). Differences were considered significant at *p* < 0.05.

## 3. Results

### 3.1. Synthesis of Endostatin 33 Peptide and Its Targeted Inhibitory Effect on Integrin α6β1 in Prostate Cancer 

**Figure 1:**
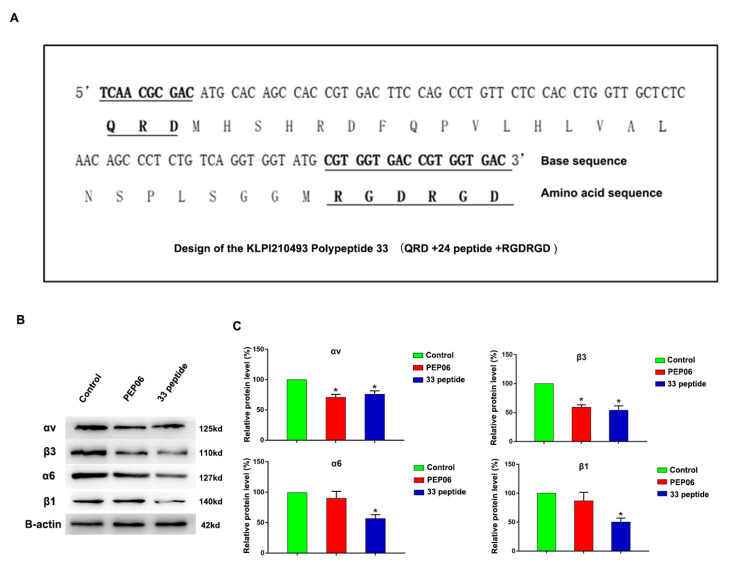
(**A**): Structure of endostatin 33 peptide (QRD (glutamine-arginine-aspartic acid) sequence + 24 peptide + RGDRGD (arginine–glycine–aspartic acid–arginine–glycine–aspartic acid) sequence). (**B**,**C**): Western blot was used to detect the protein expression of αvβ3 and α6β1 in the 33 polypeptide group, PEP06 group and 5% glucose solution group. (* stands for *p* value < 0.05).

In our previous works, we obtained PEP06 polypeptide 30 by retaining the first 24 amino acids of endostatin as the anti-angiogenic active region and adding the RGDRGD sequence to the carboxyl end of the 24 peptide. In this work, we further added the QRD sequence to the N-terminus of the PEP06 to synthesize an endostatin 33 polypeptide (Figure 1A). Therefore, the 33 polypeptide comprises the 24 peptide with anti-angiogenic activities, RGDRGD sequence and QRD sequence, of which the QRD sequence can specifically bind to α6β1. The Western blot showed that PEP06 and the 33 polypeptide could reduce the protein expression of integrin αvβ3, but the protein expression of α6β1 integrin significantly decreased only in the 33 polypeptide group (Figure 1B,C, the source data are available as supplementary Figure S1). These results suggested that peptide 33 might have a stronger antitumor effect than PEP06, especially for tumors with the high α6β1 integrin subtype, such as prostate cancer. 

### 3.2. The 33 Polypeptide Can Inhibit the Proliferation and Promote Apoptosis of PCa

**Figure 2:**
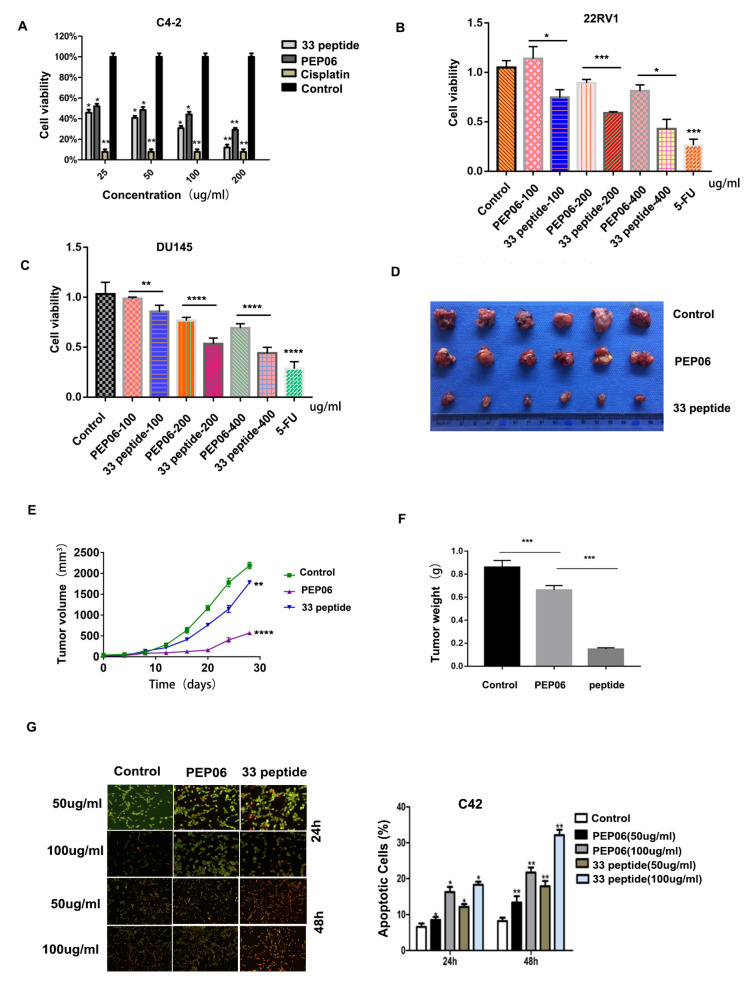
(**A**): MTT assay performed to detect the activity of C4-2 cells after 24 h treatment with 33 polypeptide (25, 50, 100 or 200 μg/mL), PEP06 (25, 50, 100 or 200 μg/mL), cisplatin (10 μg/mL) and 5% glucose solution. (**B**,**C**): MTT assay was performed to detect the activity of 22RV-1 and DU145 cells after 24 h treatment with 33 polypeptide (100, 200, or 400 μg/mL), PEP06 (100, 200 or 400 μg/mL), 5-fluorouracil (10 μg/mL) and 5% glucose solution. (**D**): Mouse subcutaneous tumorigenesis images of C4-2 cells after 24 h treatment with 33 polypeptide (50 µg/mL), PEP06 (50 µg/mL) and 5% glucose saline (n = 6 in each group). (**E**): Tumor volume measurement results of the 33 polypeptide group, PEP06 group and 5% glucose saline group. (**F**): Tumor weight measurement results of the 33 polypeptide group, PEP06 group and 5% glucose saline group, respectively. (**G**): AO/EB double fluorescent staining was used to detect the apoptosis of C4-2 cells of 33 polypeptide (50 µg/mL and 100 µg/mL) group, PEP06 (50 µg/mL and 100 µg/mL) group and 5% glucose saline group after treatment for 24 h and 48 h, respectively. (*, **, *** and **** stand for *p* value < 0.05, 0.01, 0.001 and 0.0001, separately).

As shown in Figure 2A, we found that both 33 polypeptide and PEP06 inhibited the cell viability of C4-2 cells when compared with the control group, and 33 polypeptide inhibited C4-2 cell activity in a dose-dependent manner, which was much more significant than PEP06. Similarly, 33 polypeptide had a more significant inhibitory effect of cell viability than PEP06 on 22RV1 and DU145 cells (Figure 2B,C). Subsequently, subcutaneous tumor formation in mice showed that 33 polypeptide and PEP06 inhibited the growth of PCa compared with the control group, and the effect of the 33 polypeptide group was more significant than that of the PEP06 group according to the results of tumor volume and weight (Figure 2D–F). Furthermore, the apoptosis results were confirmed by AO/EB double staining. As shown in Figure 2G, no significant apoptosis was detected in the negative control group, and early-stage apoptotic cells marked by crescent-shaped or granular yellow-green AO nuclear staining were detected in the 33 polypeptide and PEP06 group. With increasing concentrations and treatment lengths, the number of early-stage and late-stage apoptotic cells increased, and the 33 polypeptide group induced cell apoptosis more strongly than the PEP06 group. These results suggested that 33 polypeptide can inhibit the proliferation and promote the apoptosis of PCa.

### 3.3. The 33 Polypeptide Can Inhibit the Invasion and Metastasis of PCa 

**Figure 3:**
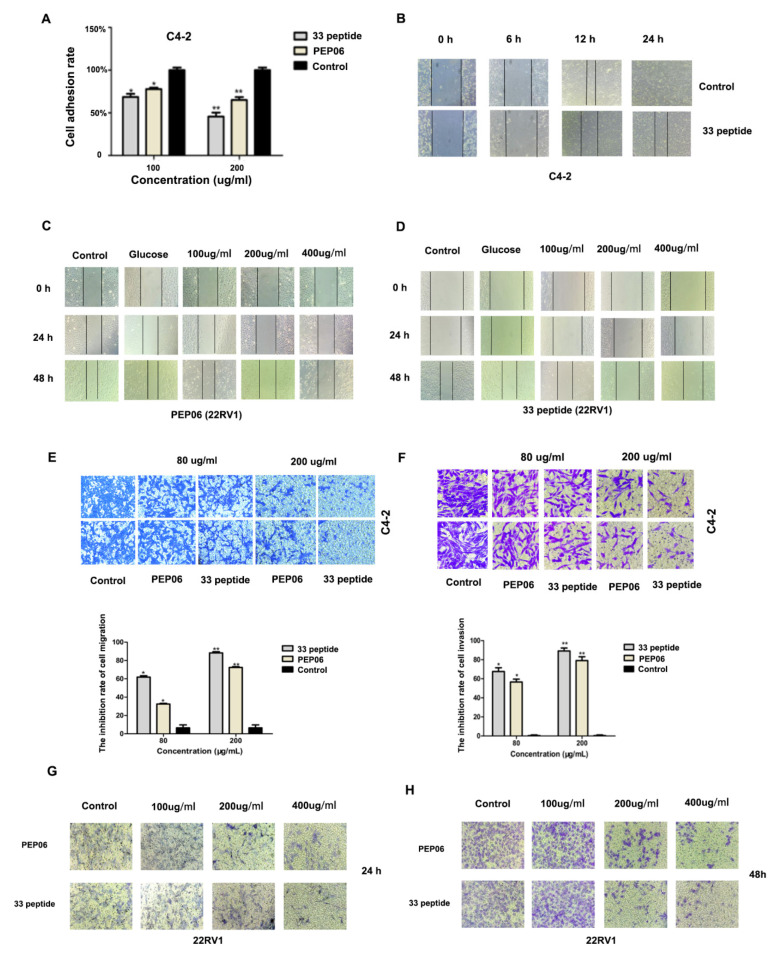
(**A**): C4-2 cell adhesion capacity was measured by cell Matrigel assay in the 33 polypeptide (100 or 200 μg/mL) treatment group, the PEP06 (100 or 200 μg/mL) treatment group and the 5% glucose saline treatment group. (**B**): Wound healing assay was performed to detect C4-2 cell migration in the 33 polypeptide (200 μg/mL) group and 5% glucose saline group after 0 h, 6 h, 12 h and 48 h treatment. (**C**): Wound healing assay was performed to detect the migration of 22RV1 cells in PEP06 group (100 µg/mL, 200 µg/mL and 400 µg/mL), 5% glucose saline group and control group after 0 h, 24 h and 48 h, respectively. (**D**): Wound healing assay was performed to detect the migration of 22RV1 cells in the 33 polypeptide group (100 µg/mL, 200 µg/mL and 400 µg/mL), 5% glucose saline group and control group after 0 h, 24 h and 48 h treatment, respectively. (**E**,**F**): Transwell assay was used to detect the migration or invasion ability of C4-2 cells in the 33 polypeptide group (80 µg/mL and 200 µg/mL), PEP06 group (80 µg/mL and 200 µg/mL) and 5% glucose saline group. (**G**,**H**): Transwell assay was performed to detect the migration of 22RV1 cells of the 33 polypeptide (100 µg/mL, 200 µg/mL and 400 µg/mL) group, PEP06 (100 µg/mL, 200 µg/mL and 400 µg/mL) group and control group for 24 h or 48 h. (* and ** stand for *p* value < 0.05 and 0.01, separately).

Next, the cell–Matrigel adhesion assay showed that the adhesion ability of C4-2 cell decreased both in the 33 polypeptide and PEP06 groups when compared with the control group, and the inhibiting adhesion ability of the 33 polypeptide group was more significant than in the PEP06 group (Figure 3A). Next, the wound healing assay showed that the 33 polypeptide significantly inhibited migration ability both in C4-2 and 22RV1 cells, and the effect of 33 peptide was more significant than that of PEP06 at 200 µg/mL and 400 µg/mL (Figure 3B–D, the source data are available as supplementary Figure S2). Furthermore, the transwell assay showed that the 33 polypeptide significantly inhibited invasion and migration abilities in C4-2 or 22RV1 cells, and the effect of 33 peptide was more significant than that of PEP06 at 200 µg/mL in C42 cells and 22RV1 cells (Figure 3E–H, the source data are available as supplementary Figure S3). These results suggested that the 33 polypeptide can inhibit the invasion and migration ability of PCa.

### 3.4. Integrin α6β1 Is Associated with Poor Prognosis and PI3K/Akt Pathway of PCa

**Figure 4:**
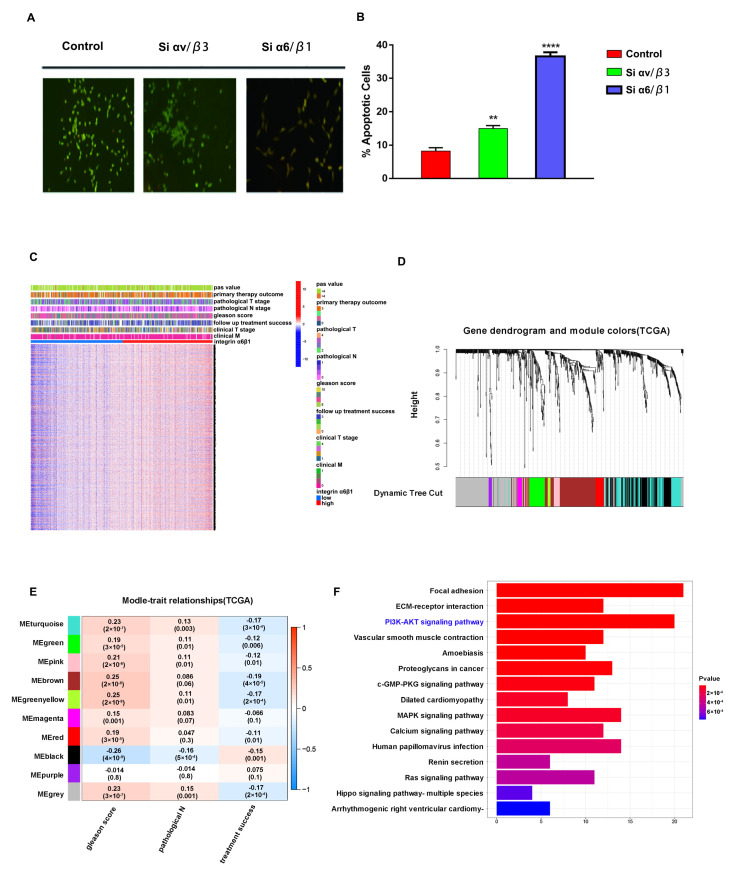
(**A**,**B**): AO/EB double fluorescent staining detected the apoptosis of C4-2 cells in the αvβ3 knockdown group, α6β1 knockdown group and 5% glucose solution group. (**C**): Heatmap of differential gene expression of integrin high-α6β1 group and integrin low-α6β1 group according to TCGA patients. (**D**,**E**): WGCNA analysis was performed to identify similar gene modules in different genes and their correlation with clinical traits (Gleason score, pathological N stage, etc.). (**F**): KEGG enrichment analysis was performed for gene modules (MEgreenyellow and MEbrown). (** and **** stand for *p* value < 0.01 and 0.0001, separately).

Firstly, αvβ3 and α6β1 were knocked down in C42 cell lines. The results showed that both the αvβ3 and α6β1 knockdown groups promoted the apoptosis of PCa cells compared with the control group, but the effect of the α6β1 knockdown group was more significant (Figure 4A,B). Next, we divided 489 PCa patients from the TCGA database into the high-α6β1 group and low-α6β1 group according to the mean value of integrin α6β1 gene expression and identified 2124 differentially expressed genes between the 2 groups (Figure 4C). Then, we performed WGCNA analyses on these differentially expressed genes and found that these genes could be aggregated into ten gene modules (Figure 4D), among which the MEbrown and Megreenyellow modules had the strongest correlation with the Gleason score (*p* < 0.05) (Figure 4E). Next, we performed KEGG analysis on 217 genes in the MEbrown and MEgreenyellow modules, and the results showed that these genes were mainly enriched in the PI3K-Akt pathway (Figure 4F). These results suggested that integrin α6β1 is closely associated with poor prognosis and the PI3K-Akt pathway of prostate cancer. Due to the targeted inhibitory effect of the 33 peptide on α6β1, it is possible that the 33 peptide may exert an anti-tumor effect by mediating integrin α6β1to inhibit the PI3K-Akt pathway.

### 3.5. The 33 Polypeptide Inhibits EMT and MMPs in PCa by Blocking the PI3K/AKT Pathway

**Figure 5:**
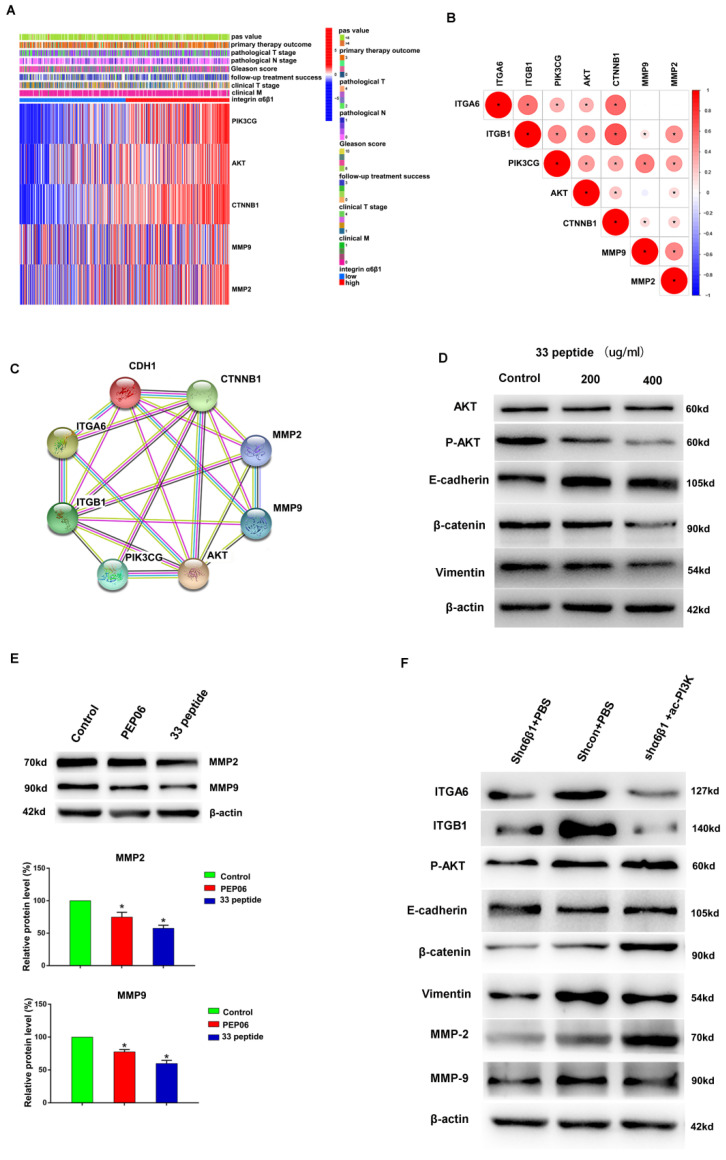
(**A**): PIK3CG, AKT, CTNNB1, MMP9 and MMP2 differentially expressed heatmap among the integrin α6β1-high group and integrin α6β1-low group. (**B**): Correlation analysis of ITGA6, ITGB1, PIK3CG, AKT, CTNNB1, MMP9 and MMP2. (**C**): PPI analysis of ITGA6, ITGB1, PIK3CG, AKT, CTNNB1, MMP9 and MMP2. (**D**): Western blot was used to detect the AKT, P-Akt, E-cadherin, β-catenin and Vimentin protein expression of different concentrations of the endostatin 33 peptide (200 µg/mL and 400 µg/mL) and 5% glucose solution groups in C4-2 cells. (**E**): Western blot was used to detect the MMP2 and MMP9 protein expression of the 33 polypeptide group, PEP06 group and 5% glucose solution group in C4-2 cells, respectively. (**F**): Western blot was used to detect the ITGA6, ITGB1, P-AKT, E-cadherin, β-catenin, Vimentin, MMP9 and MMP2 of the integrin α6β1 knockdown group, integrin α6β1 knockdown +PI3K activation group and control group in C4-2 cells. (* stands for *p* value < 0.05).

Firstly, the α6β1-high group was found to have higher expression levels of PI3K, AKT, β-catenin (CTNNB1, which is closely related to the development of EMT), MMP2 and MMP9 in 489 PCa cases from the TCGA database. Meanwhile, ITGA6, ITGB1, PI3K, AKT, β-catenin, MMP2 and MMP9 showed a moderate- to high-intensity positive correlation (*p* < 0.05) (Figure 5A,B). Similarly, the PPI network further verified good protein interactions and potential targeted regulatory relationships among ITGA6, ITGB1, PI3K, AKT, β-catenin, E-cadherin (CDH1), MMP2 and MMP9 (Figure 5C). These results suggested a close association between integrin α6β1 and PI3K-Akt, EMT and MMPs. Since peptide 33 has a targeted inhibitory effect on integrin α6β1, we hypothesized that peptide 33 might exert anti-tumor effect by inhibiting PI3K-Akt, EMT and MMPs. Then, we added different concentrations of the endostatin 33 peptide (200 µg/mL and 400 µg/mL) into C42 cells, and the results showed that the protein expression levels of P-Akt, β-catenin and vimentin were significantly inhibited, e-cadherin was promoted, and total AKT was not affected when compared with the control group in different concentrations (Figure 5D, the source data are available as supplementary Figure S1). In addition, endostatin 33 peptide significantly inhibited MMP2 and MMP-9 protein levels in C42 cells when compared with the control group and PEP06 group (Figure 5E, the source data are available as supplementary Figure S1). These results further demonstrated the regulatory effect of endostatin 33 peptide on PI3K-Akt, EMT and MMPs. To further confirm the specific regulatory relationship between integrin α6β1, P-Akt, EMT and MMPs, we knocked down the expression level of integrin α6β1 in C42 cells; the results showed that the expression level of P-Akt in the α6β1 knockdown group was significantly down-regulated compared with the control group, and the expression levels of EMT protein and MMPs were synchronously inhibited (Figure 5F, the source data are available as supplementary Figure S1). Next, we activated the PI3K pathway on the basis of α6β1 knockdown, and the results showed that the expression of P-Akt was upregulated compared with the α6β1 knockdown group, and EMT protein and MMPs were simultaneously increased, while the expression of integrin α6β1 was not affected (Figure 5F, the source data are available as supplementary Figure S1). These results suggested that endostatin 33 peptide inhibits the PI3K-Akt pathway via the targeted inhibition of integrin α6β1, and the inhibition of the PI3K-Akt pathway in turn inhibits EMT and MMPs expression, thus exerting an anti-tumor biological effect in PCa.

## 4. Discussion

In the present study, endostatin 33 peptide contains the arginine–glycine–aspartic acid (RGD) and glutamine–arginine–aspartic acid (QRD) sequences and 24 peptide antiangiogenic regions. The RGD sequence is considered to be the cell attachment site of the adhesion protein [22], and it can be recognized by the highly expressed heterodimer αvβ3 integrin in tumor cells [5]. Integrin α6β1 is a subclass that cannot be recognized by the RGD sequence and is involved in CRPC resistance [23]. CD151 is a cell surface protein that belongs to the tetraspan superfamily of transmembrane proteins, which forms a functional complex with integrins to mediate cell migration, signal transduction and angiogenesis [24]. The QRD sequence is a specific site of the extracellular large loop of CD151, which can specifically recognize and interact with α6β1 integrin [20,21], causing downstream signal activation. Therefore, endostatin 33 peptide can bind to both integrin αvβ3 and α6β1 to improve the antitumor activity, especially for cancers with a high expression of integrin α6β1 or cancers that are not recognized by RGD, as we found that the anti-tumor effect of endostatin 33 peptide on PCa with a high expression of integrin α6β1 was significantly better than PEP06.

Tumor metastasis is a multistep process by which cancer cells from the primary tumor enter the vasculature and circulate, migrate and invade distant secondary organs or tissues. This cascaded development requires signal communication at each step, which occurs within cells, between cells or between cells and the extracellular matrix (ECM) [25]. The adhesion of tumor cells is generally regarded as the first step of the entire process, and the breakdown of the basement membrane is considered to be a critical step in the metastasis process. In this study, endostatin 33 peptide significantly inhibited cell adhesion to the Matrigel matrix in a concentration-dependent manner. Additionally, in a Matrigel-based invasion model, we found that the number of invading cells in the endostatin 33 peptide group at the same concentration was significantly less than that in the PEP06 group. 

It was reported that the Phosphoinositol 3 kinase/protein kinase B (PI3-K/AKT) signaling pathway plays an important role in the progression of castration-resistant prostate cancer (CRPC) and is often activated in regulating integrin-mediated cell migration [26,27]. For integrin α6β1, it seems to be closely related to the activation of the PI3K/AKT pathway. It has been reported that integrin α6β1 activated the PI3K/AKT pathway and contributed to epithelial–mesenchymal transition (EMT) in cholangiocarcinoma (CCA) [28], while it has not been fully elucidated in PCa. Surprisingly, our study demonstrated that endostatin 33 peptide can block the PI3K-Akt pathway by targeting the inhibition of integrin α6β1, thus playing an anti-tumor role in PCa.

Many mechanisms facilitate the progression of prostate cancer to CRPC. Matrix metalloproteinases (MMPs) belong to a large family capable of degrading most components of the extracellular matrix (ECM), affecting basic cellular processes, including cell proliferation, invasion, metastasis and angiogenesis, especially MMP-2 and MMP-9 [28,29]. Integrins have been reported to play a crucial role in the activation of these pathways or proteases, especially αvβ3 and α6β1 [30,31], but their specific mechanism in PCa has not been fully understood. Furthermore, the epithelial–mesenchymal transition (EMT) is a critical mechanism for many pathological processes, including tumor invasion and metastasis [32]. Our study also showed a close association between endostatin 33 peptide and EMT and MMPs. Specifically, we found that endostatin 33 peptide significantly inhibited the MMPs and EMT, and this process is mainly achieved by regulating the PI3K-Akt pathway.

Taken together, our data suggested that the endostatin 33 peptide containing QRD sequences may potently suppress the metastasis, invasion and proliferation of the PCa. The antitumor effect of the endostatin 33 peptide is more obvious than that of the PEP06, and its antitumor mechanism may involve the PI3-K/AKT signaling pathway, which is mediated by α6β1 integrin. 

## 5. Conclusions

In conclusion, our study developed a novel 33 polypeptide that can specifically identify high-expression α6β1-subtype tumors and that has stronger antitumor activity than the existing PEP06. In addition, endostatin 33 peptide mainly inhibits EMT and MMPs by inhibiting the PI3K-Akt pathway, thus exerting an anti-tumor effect in PCa. Our study will provide a new solution and theoretical basis for the treatment of PCa.

## Data Availability

All data in this study are available at the TCGA data portal (https://tcga-data.nci.nih.gov/tcga/, accessed on 1 February 2022) and this article.

## Supplementary Materials

The following supporting information can be downloaded at: https://www.mdpi.com/2077-0383/12/5/1861/s1, Figure S1: The source data of Figure 1B and Figure 5D–F; Figure S2: The source data of Figure 3B–D; Figure S3: The source data of Figure 3E–H.

## Abbreviations

PCa: Prostate cancer; RGD: arginine–glycine–aspartic acid; QRD: glutamine–arginine–aspartic cid; PEP06: endostatin 30 peptide; EMT: epithelial–mesenchymal transition; MMPs: matrix metalloproteinase; CRPC: castration-resistant prostate cancer; ECM: extracellular matrix; TCGA: The Cancer Genome Atlas; WGCNA: weighted gene co-expression network analysis; KEGG: Kyoto Encyclopedia of Genes and Genomes; PPI: protein–protein interaction; AO/EB: acridine orange and ethidium bromide; FU: 5-fluorouracil; SH, Si: gene knockdown group; ac-PI3K: PI3K activation group.
