# Correction: Liu et al. Endostatin 33 Peptide Is a Deintegrin α6β1 Agent That Exerts Antitumor Activity by Inhibiting the PI3K-Akt Signaling Pathway in Prostate Cancer. *J. Clin. Med.* 2023, *12*, 1861

**DOI:** 10.3390/jcm13133943

**Published:** 2024-07-05

**Authors:** Yang Liu, Chang-Lin Wang, Zhong-Qi Pang, Ke Gao, Lin-Kun Shen, Wan-Hai Xu, Ming-Hua Ren

**Affiliations:** 1 Department of Urology, The First Affiliated Hospital of Harbin Medical University, Harbin 150001, China; 202101233@hrbmu.edu.cn (Y.L.); 2021020849@hrbmu.edu.cn (Z.-Q.P.); 2022020675@hrbmu.edu.cn (K.G.); 2020020828@hrbmu.edu.cn (L.-K.S.); 2 Department of Urology, The Fourth Affiliated Hospital of Harbin Medical University, Harbin 150001, China; 601740@hrbmu.edu.cn

In the original publication [1], there was a mistake in Figure 5 and Figure S1 as published. The authors found an inconsistency between the image labeled “MMP2” in Figure 5E of the article and the records in the original experimental notebook; to ensure the accuracy of the result, Figure 5E needs to be updated. Since the image of “MMP2” in Figure S1 corresponds to Figure 5E, the image of “MMP2” in Figure S1 needs to be updated simultaneously. The corrected Figure 5 appears below. The authors state that the scientific conclusions are unaffected. This correction was approved by the Academic Editor. The original publication has also been updated.

## Figures and Tables

**Figure 5:**
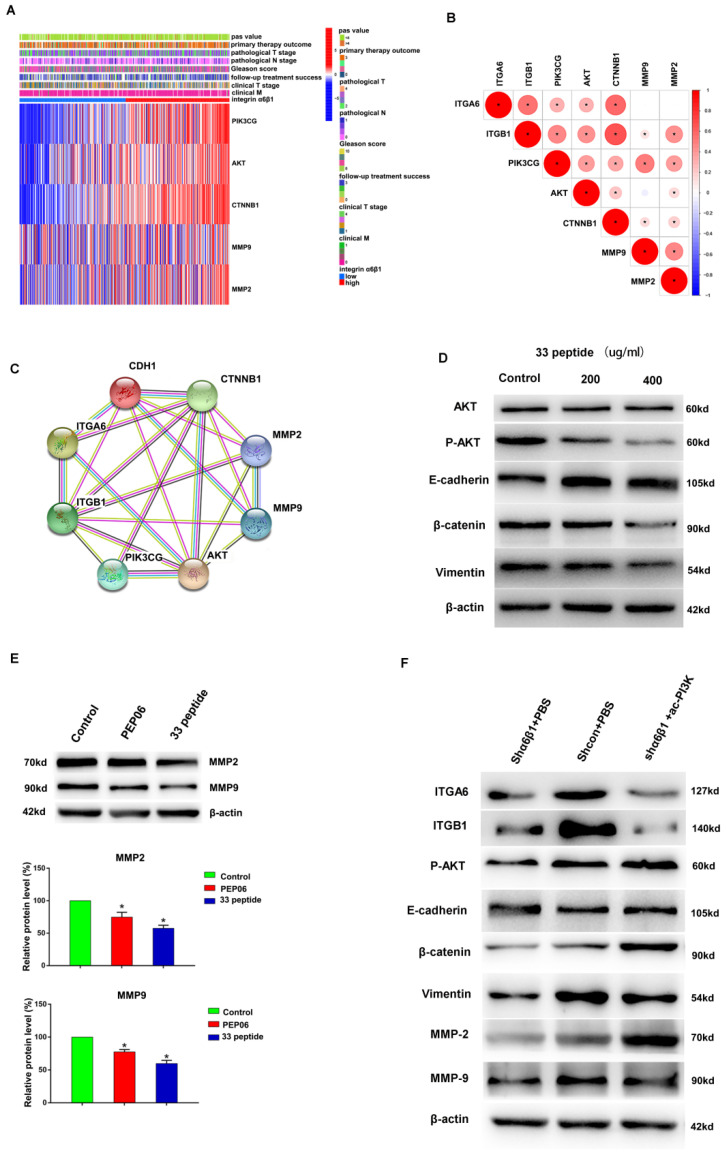
(**A**): PIK3CG, AKT, CTNNB1, MMP9 and MMP2 differentially expressed heatmap among the integrin α6β1-high group and integrin α6β1-low group. (**B**): Correlation analysis of ITGA6, ITGB1, PIK3CG, AKT, CTNNB1, MMP9 and MMP2. (**C**): PPI analysis of ITGA6, ITGB1, PIK3CG, AKT, CTNNB1, MMP9 and MMP2. (**D**): Western blot was used to detect the AKT, P-Akt, E-cadherin, β-catenin and Vimentin protein expression of different concentrations of the endostatin 33 peptide (200 µg/mL and 400 µg/mL) and 5% glucose solution groups in C4-2 cells. (**E**): Western blot was used to detect the MMP2 and MMP9 protein expression of the 33 polypeptide group, PEP06 group and 5% glucose solution group in C4-2 cells, respectively. (**F**): Western blot was used to detect the ITGA6, ITGB1, P-AKT, E-cadherin, β-catenin, Vimentin, MMP9 and MMP2 of the integrin α6β1 knockdown group, integrin α6β1 knockdown +PI3K activation group and control group in C4-2 cells. (* stands for *p* value < 0.05).
