# Supplementary material for: Endostatin 33 Peptide Is a Deintegrin α6β1 Agent That Exerts Antitumor Activity by Inhibiting the PI3K-Akt Signaling Pathway in Prostate Cancer
Source: J Clin Med. 2023 Feb 27;12(5):1861. doi: 10.3390/jcm12051861 (PMC10003382; doi:10.3390/jcm12051861)
Supplement: Supplementary file 1 [file jcm-12-01861-s001.zip › Supplymentary Figure S2.pdf]

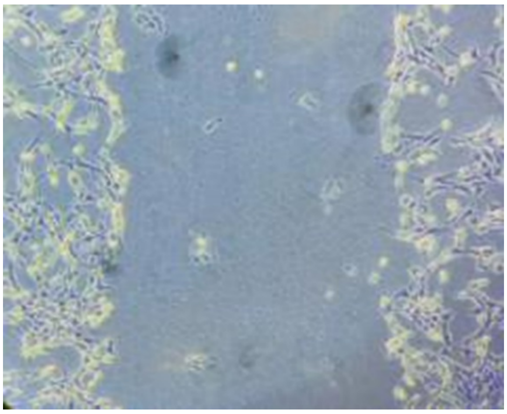

Figure S2 Control group 0h

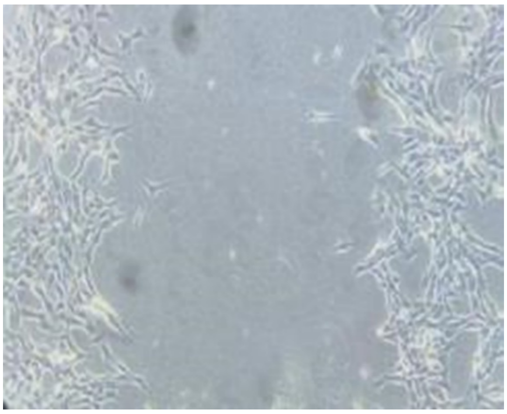

Figure S2 Control group 6h

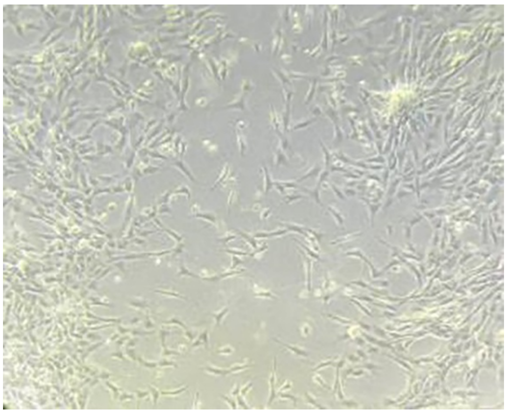

Figure S2 Control group 12h

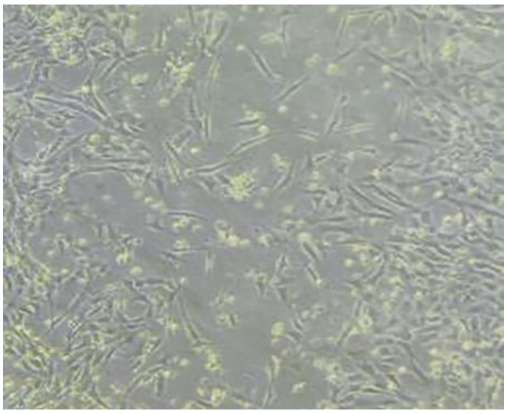

Figure S2 Control group 24h

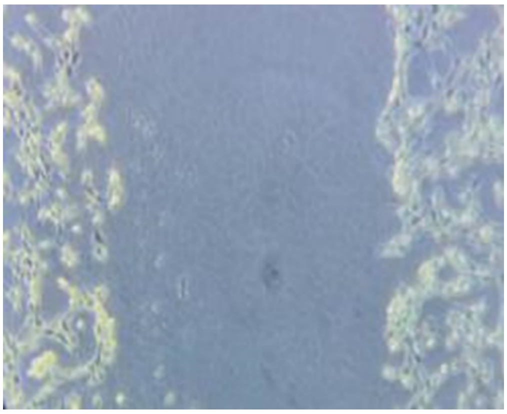

Figure S2 33-peptide group 0h

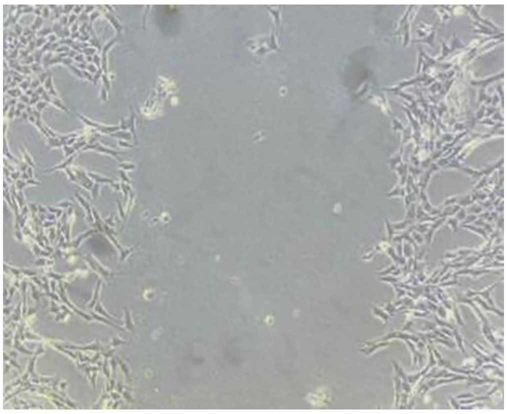

Figure S2 33-peptide group 6h

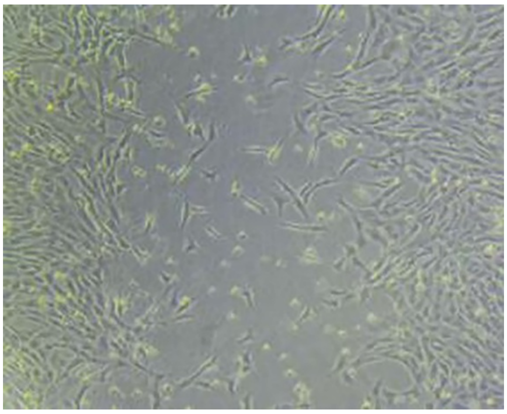

Figure S2 33-peptide group 12h

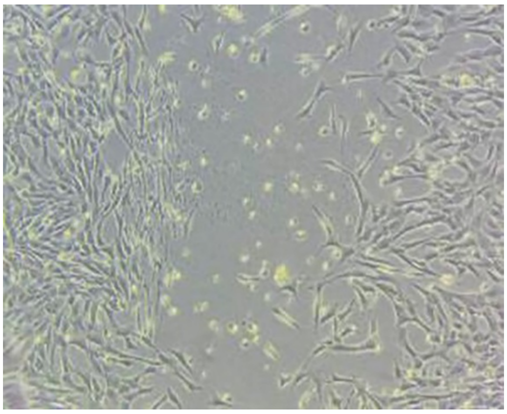

Figure S2 33-peptide group 24h

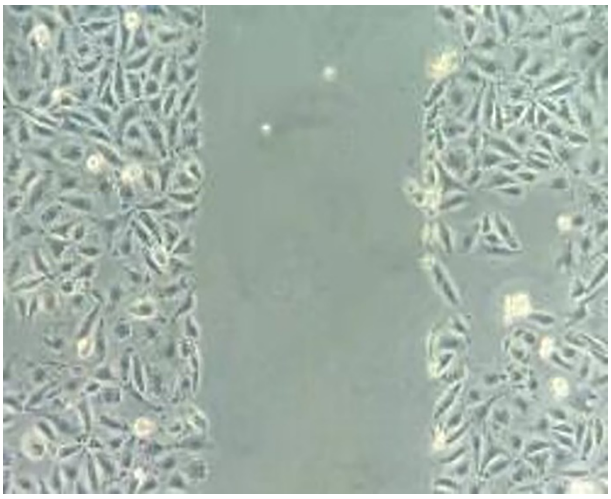
Figure S2 Control group 0h

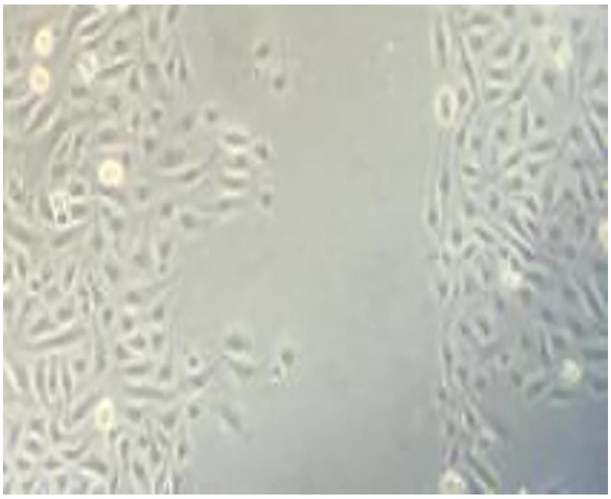
Figure S2 Control group 24h

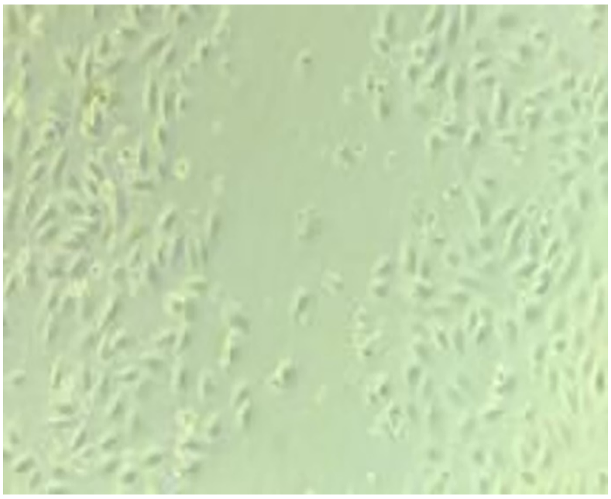
Figure S2 Control group 48h

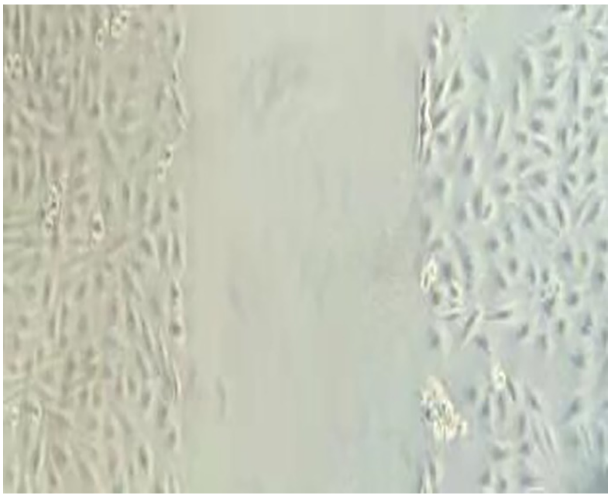
Figure S2 Glucose treating group 0h

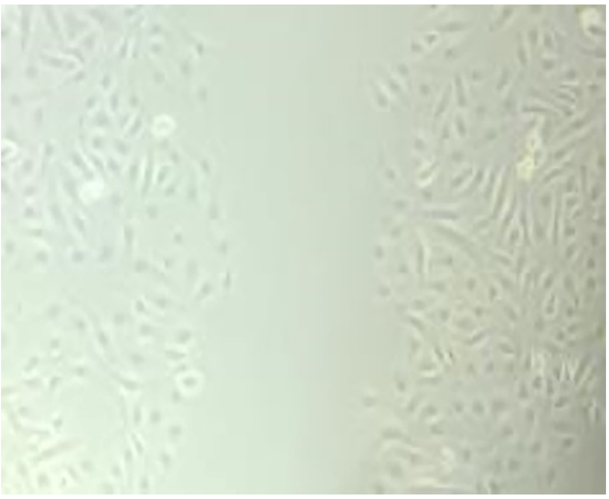
Figure S2 Glucose treating group 24h

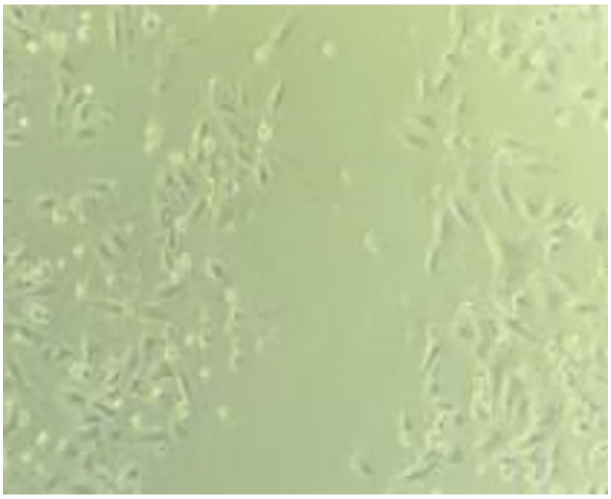
Figure S2 Glucose treating group 48h

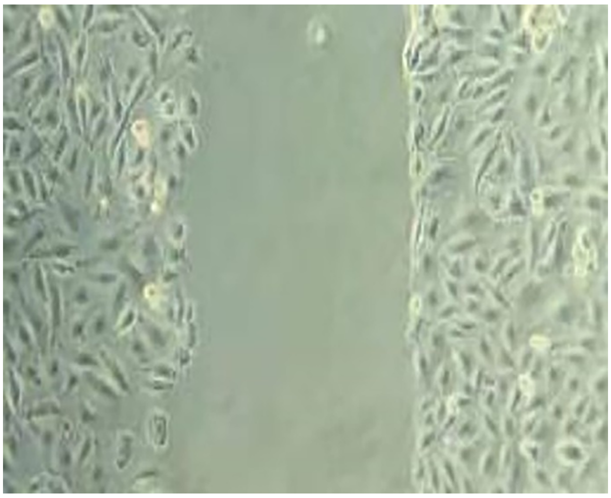
Figure S2 30-peptide 100ug/ml treating group 0h

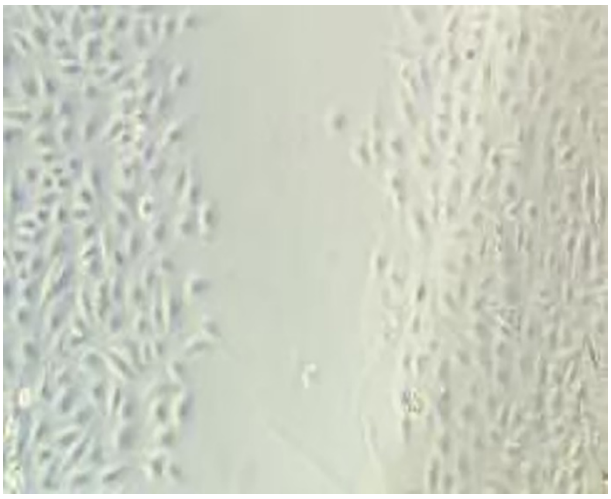
Figure S2 30-peptide 100ug/ml treating group 24h

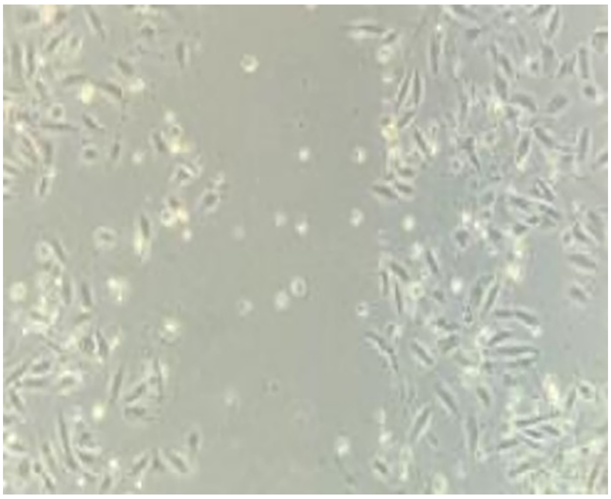
Figure S2 30-peptide 100ug/ml treating group 48h

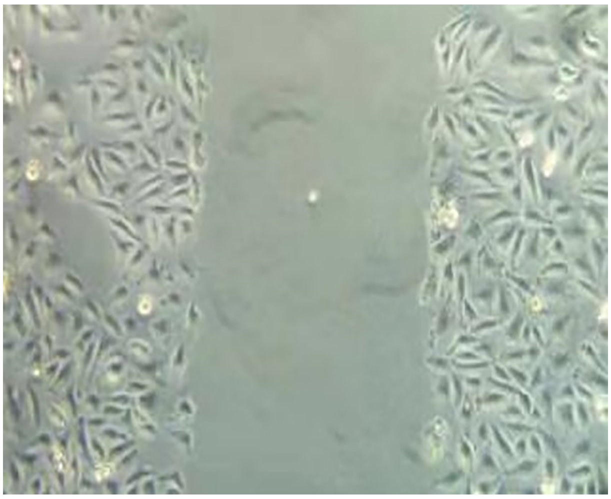

Figure S2 30-peptide 200ug/ml treating group 0h

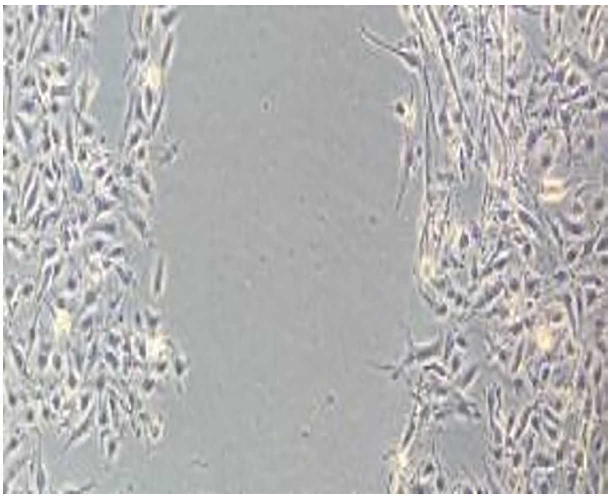

Figure S2 30-peptide 200ug/ml treating group 24h

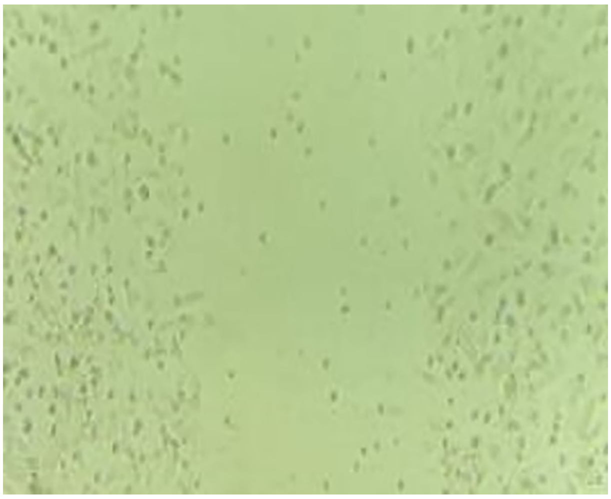

Figure S2 30-peptide 200ug/ml treating group 48h

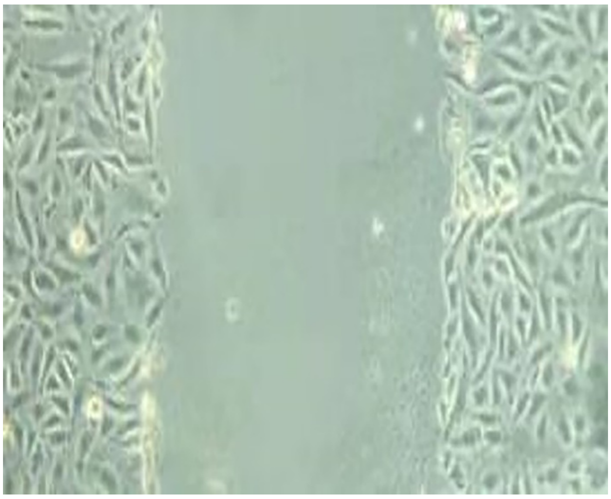

Figure S2 30-peptide 400ug/ml treating group 0h

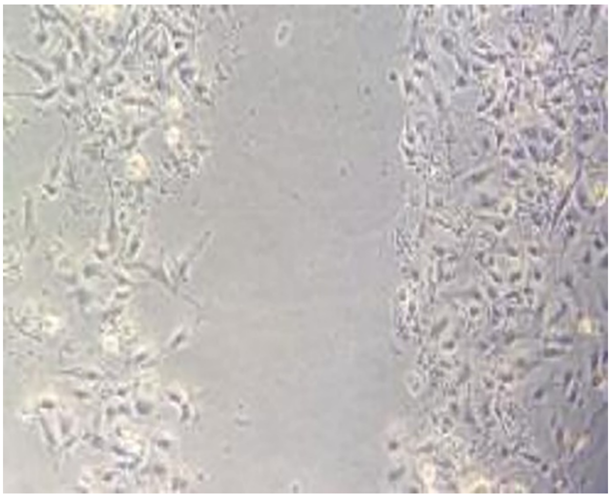

Figure S2 30-peptide 400ug/ml treating group 24h

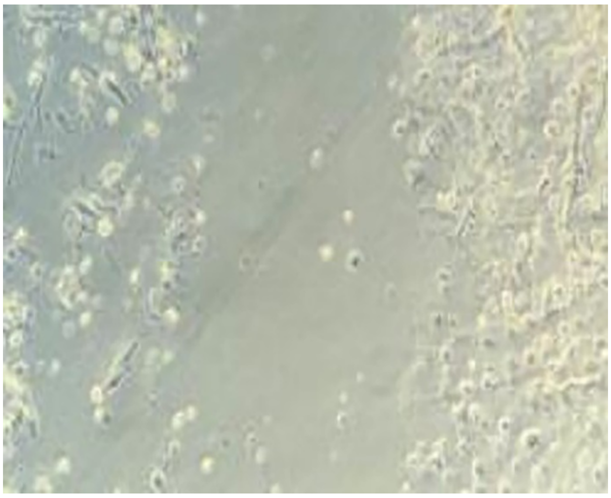

Figure S2 30-peptide 400ug/ml treating group 48h

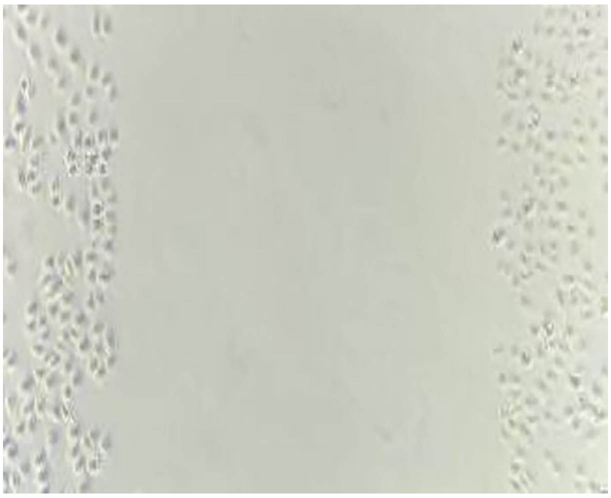

Figure S2 Control group 0h

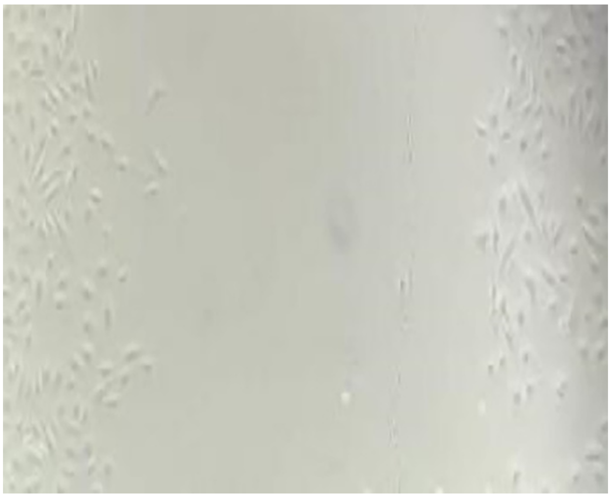

Figure S2 Control group 24h

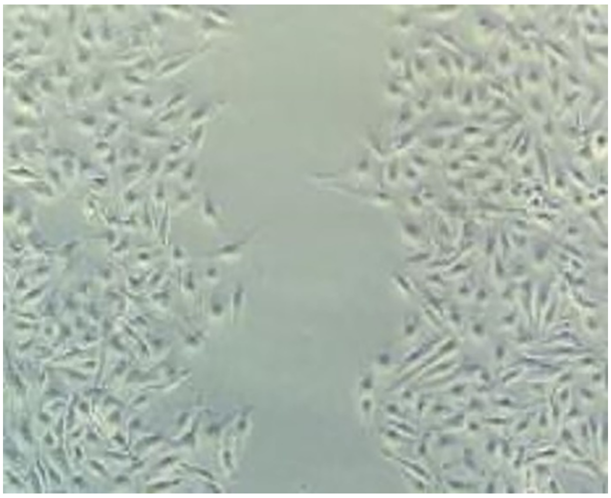

Figure S2 Control group 48h

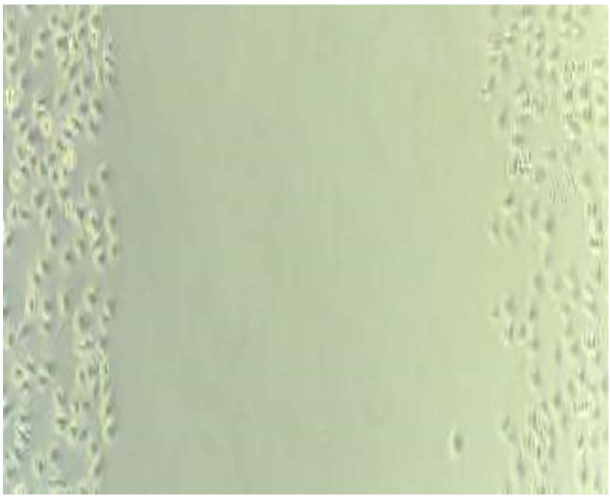

Figure S2 Glucose treating group 0h

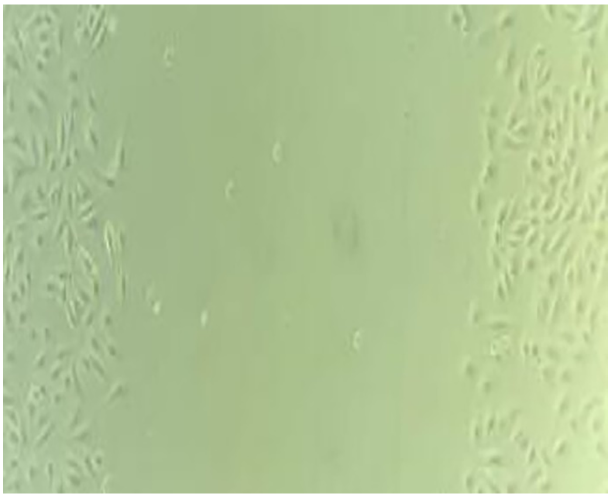

Figure S2 Glucose treating group 24h

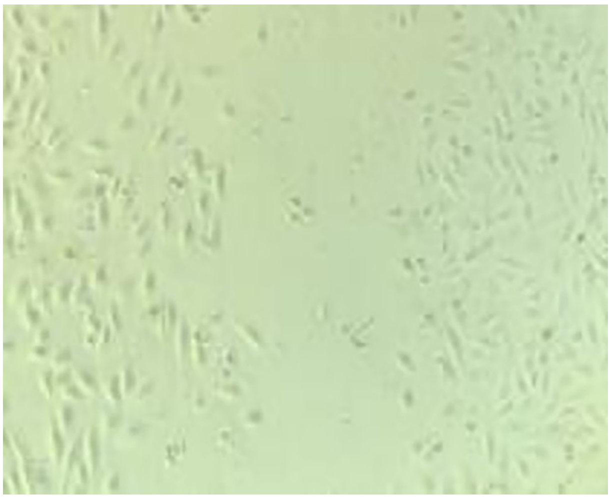

Figure S2 Glucose treating group 48h

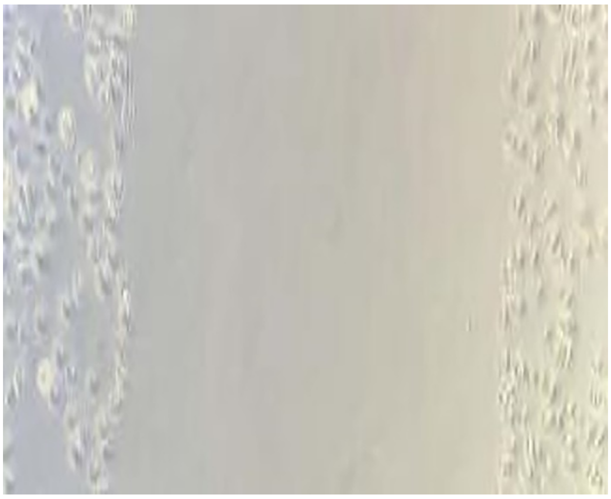

Figure S2 33-peptide 100 ug/ml treating group 0h

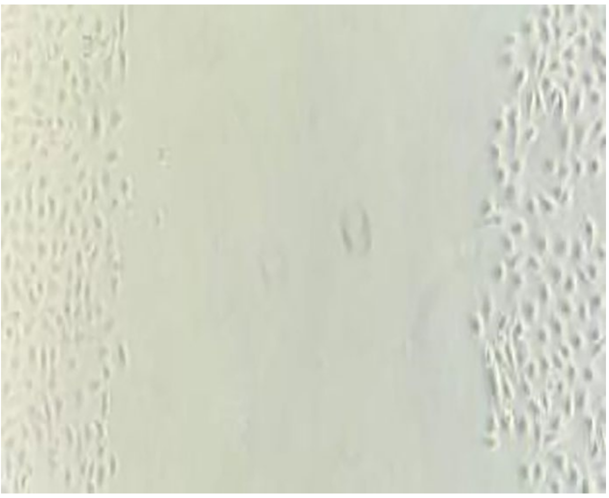

Figure S2 33-peptide 100 ug/ml treating group 24h

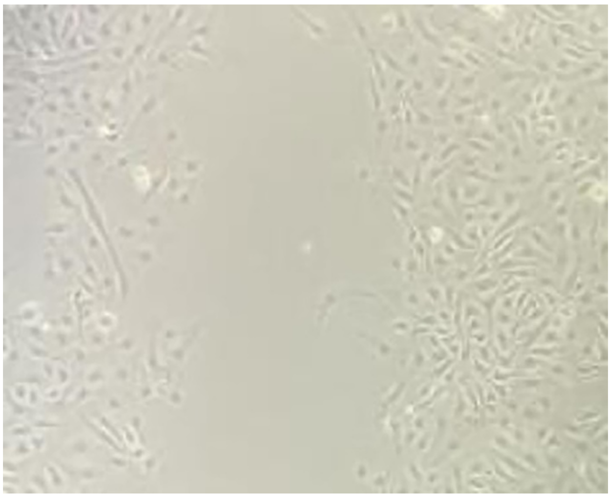

Figure S2 33-peptide 100 ug/ml treating group 48h

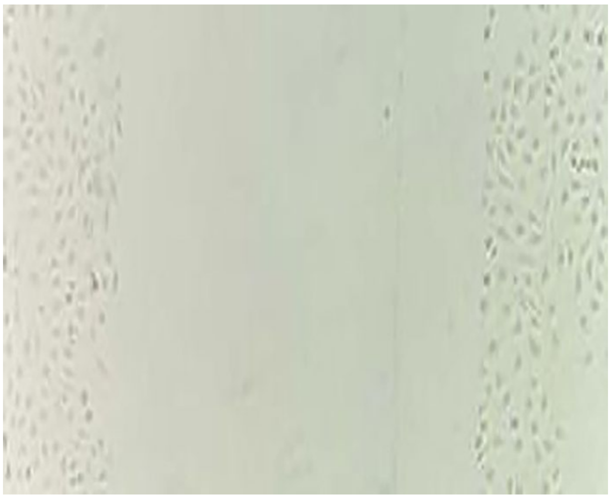

Figure S2 33-peptide 200 ug/ml treating group 0h

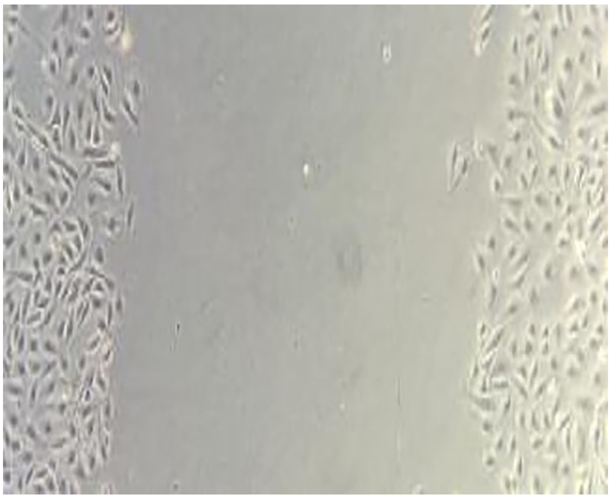

Figure S2 33-peptide 200 ug/ml treating group 24h

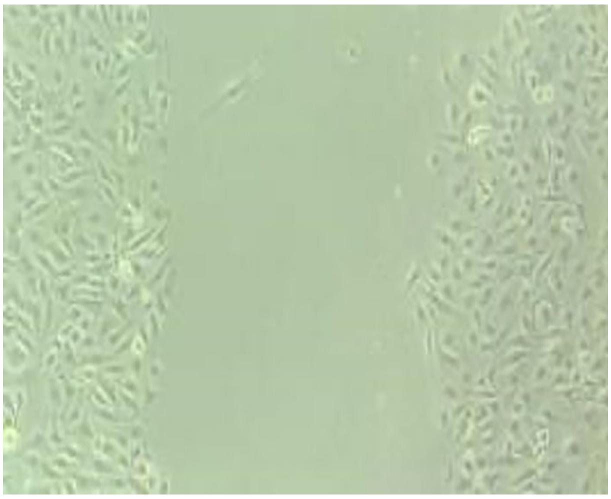

Figure S2 33-peptide 200 ug/ml treating group 48h

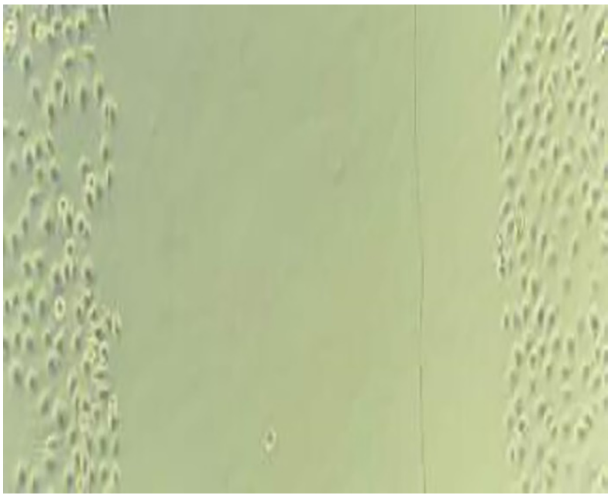

Figure S2 33-peptide 400 ug/ml treating group 0h

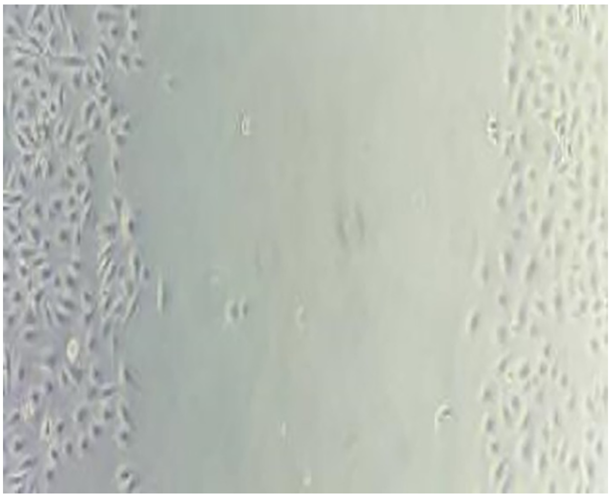

Figure S2 33-peptide 400 ug/ml treating group 24h

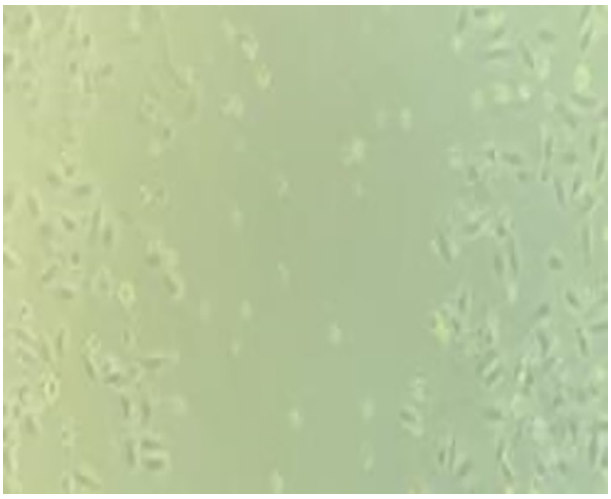

Figure S2 33-peptide 400 ug/ml treating group 48h
