# Supplementary material for: Endostatin 33 Peptide Is a Deintegrin α6β1 Agent That Exerts Antitumor Activity by Inhibiting the PI3K-Akt Signaling Pathway in Prostate Cancer
Source: J Clin Med. 2023 Feb 27;12(5):1861. doi: 10.3390/jcm12051861 (PMC10003382; doi:10.3390/jcm12051861)
Supplement: Supplementary file 1 [file jcm-12-01861-s001.zip › Supplymentary Figure S3.pdf]

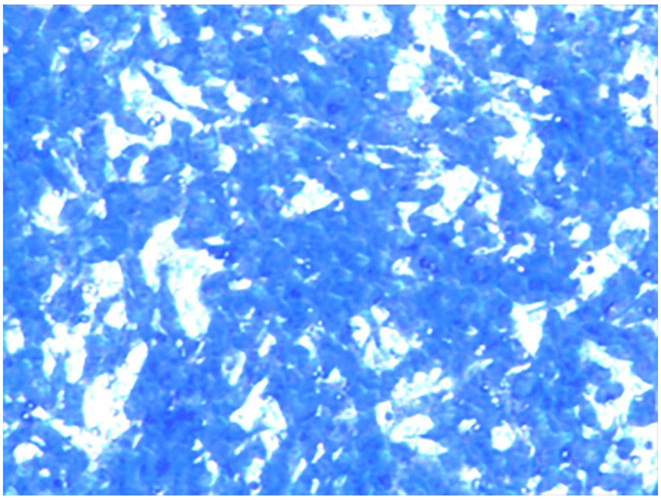

Figure S3 Control group picture 1

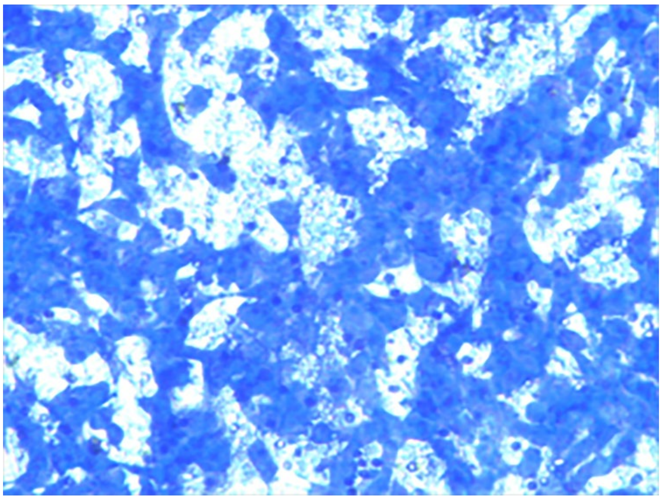

Figure S3 Control group picture 2

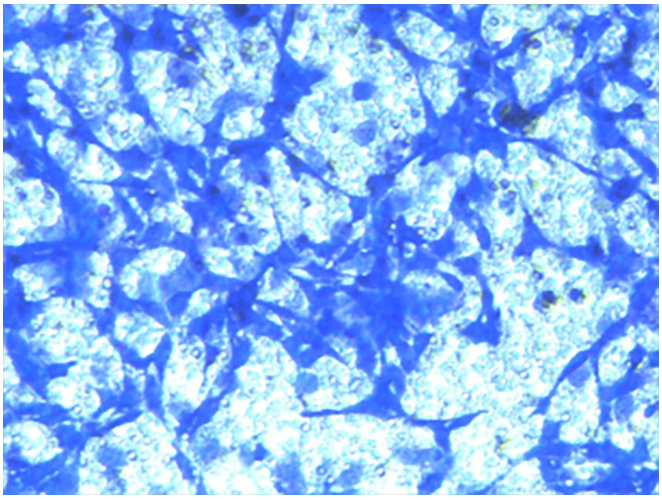

Figure S3 33-peptide 80ug/ml group Picture 1

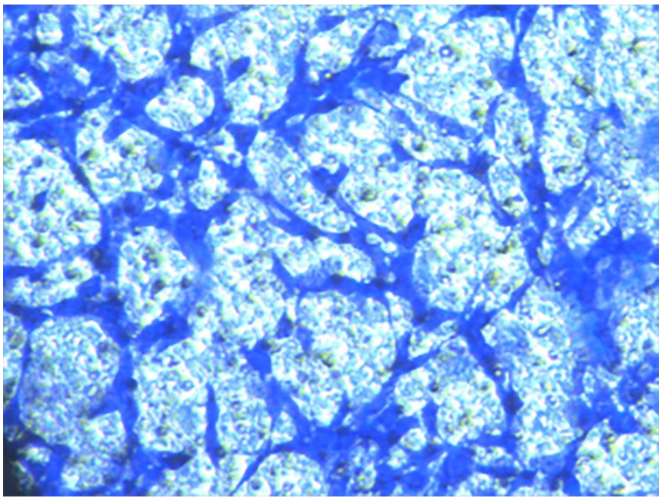

Figure S3 33-peptide 80ug/ml group Picture 2

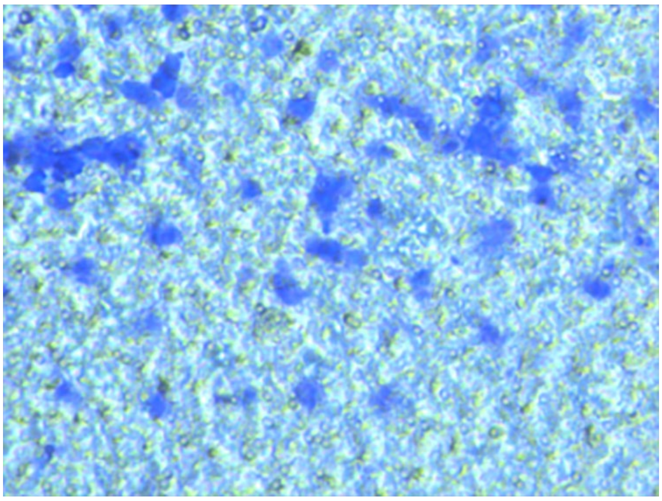

Figure S3 33-peptide 200ug/ml group Picture 1

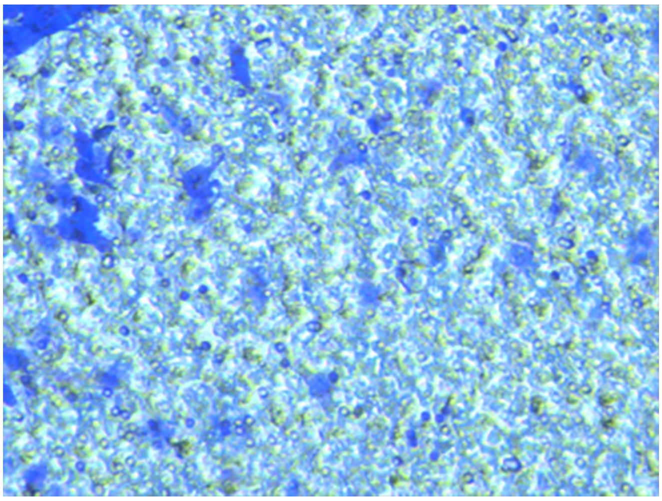

Figure S3 33-peptide 200ug/ml group Picture 2

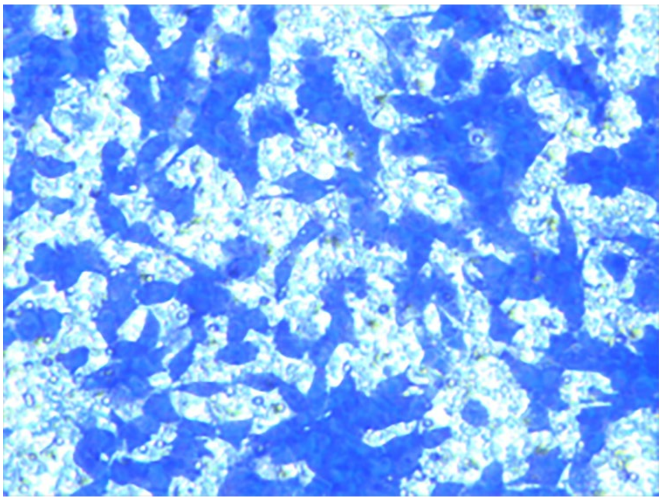

Figure S3 30-peptide 80ug/ml group Picture 1

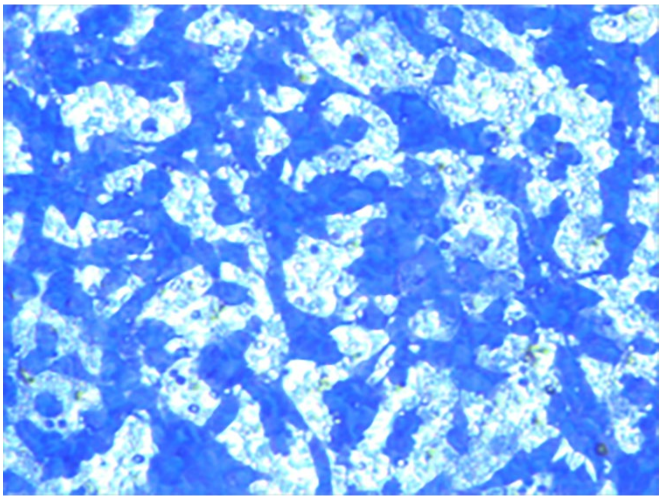

Figure S3 30-peptide 80ug/ml group Picture 2

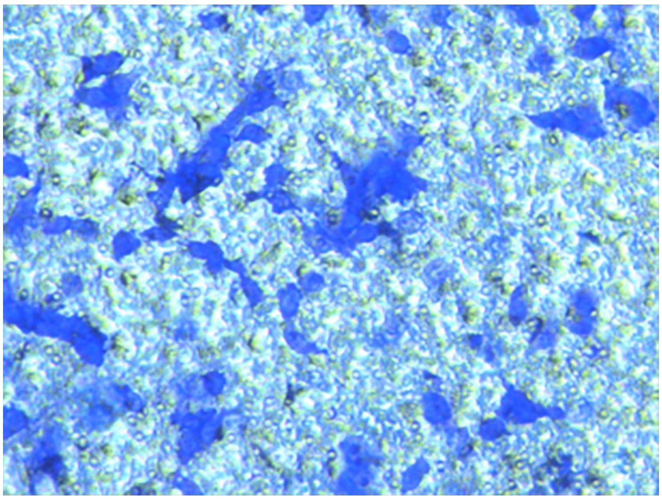

Figure S3 30-peptide 200ug/ml group Picture 1

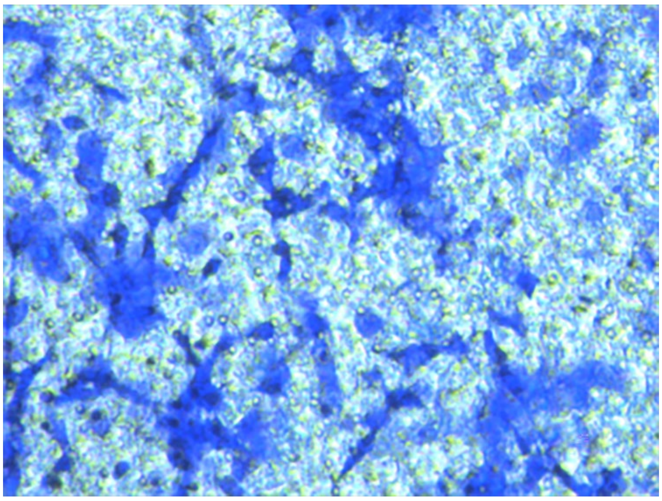

Figure S3 30-peptide 200ug/ml group Picture 2

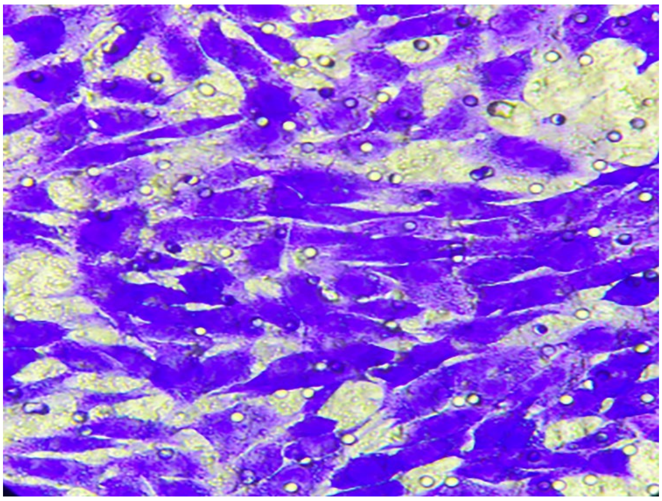

Figure S3 Control group Picture 1

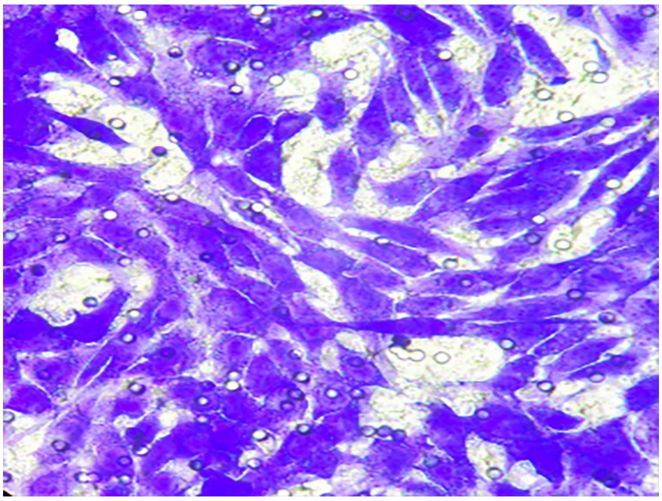

Figure S3 Control group Picture 2

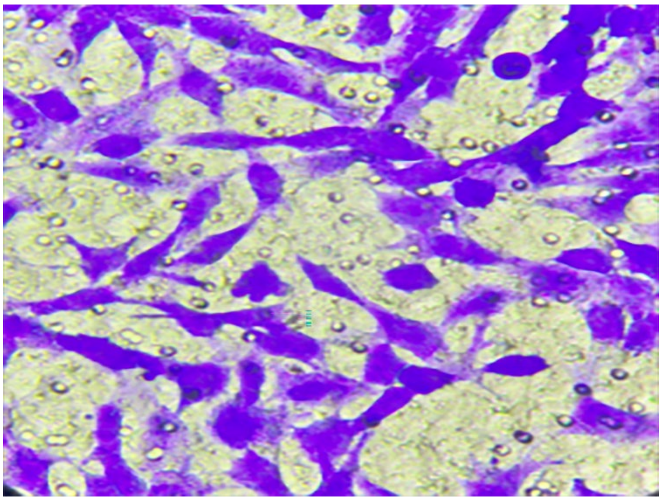

Figure S3 33-peptide 80ug/ml group Picture 1

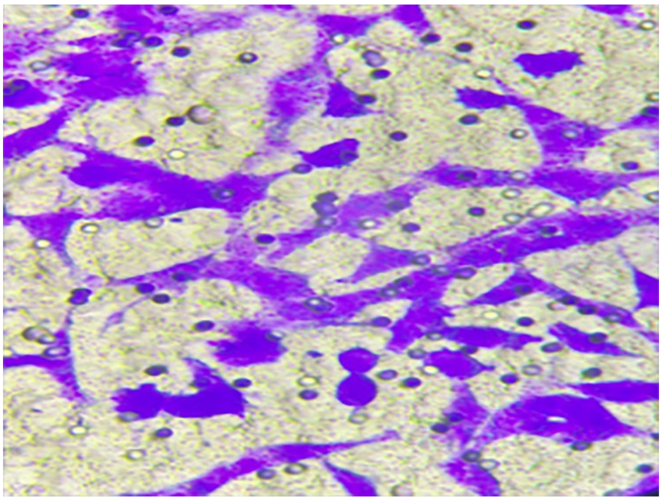

Figure S3 33-peptide 80ug/ml group Picture 2

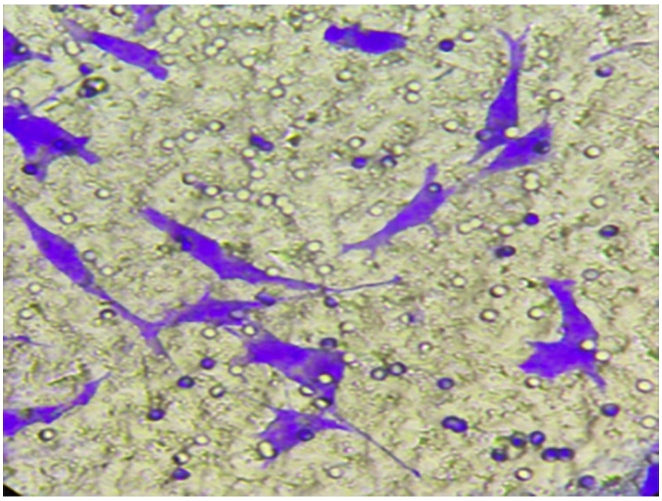

Figure S3 33-peptide 200ug/ml group Picture 1

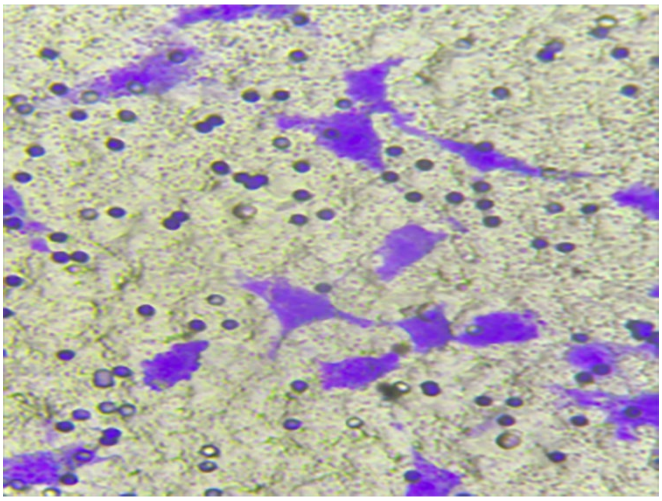

Figure S3 33-peptide 200ug/ml group Picture 2

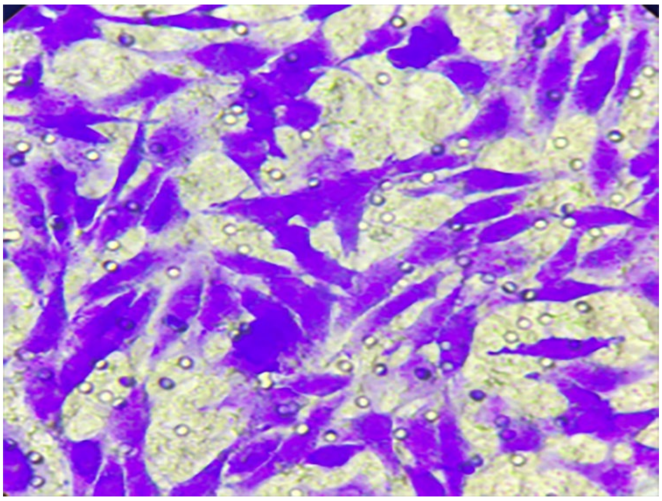

Figure S3 30-peptide 80ug/ml group Picture 1

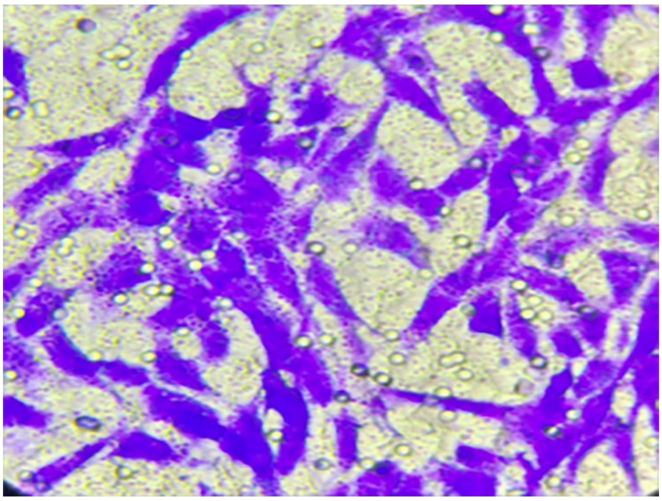

Figure S3 30-peptide 80ug/ml group Picture 2

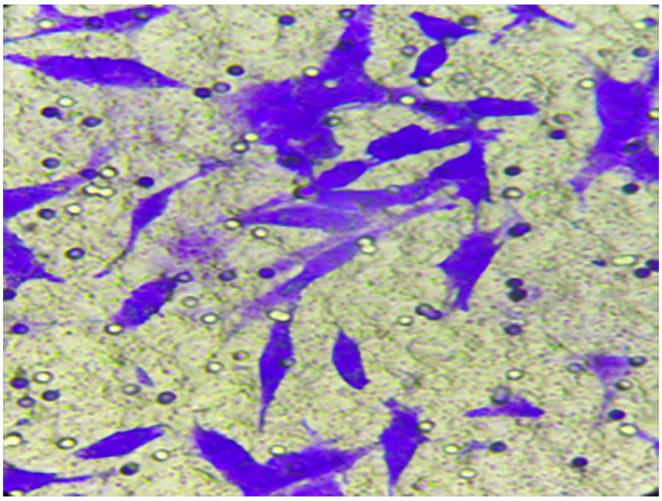

Figure S3 30-peptide 200ug/ml group Picture 1

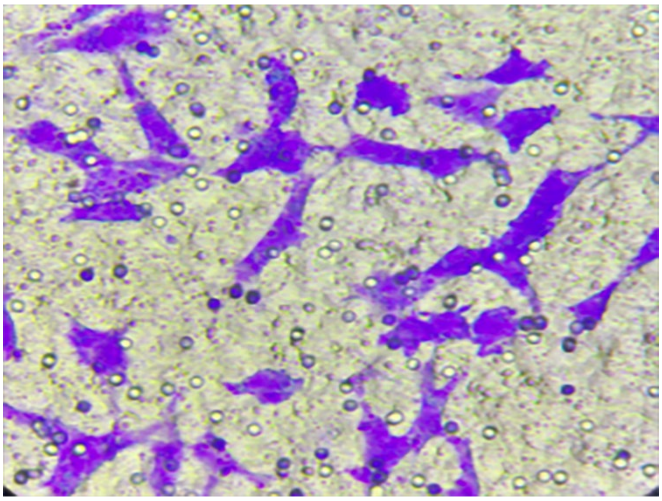

Figure S3 30-peptide 200ug/ml group Picture 2

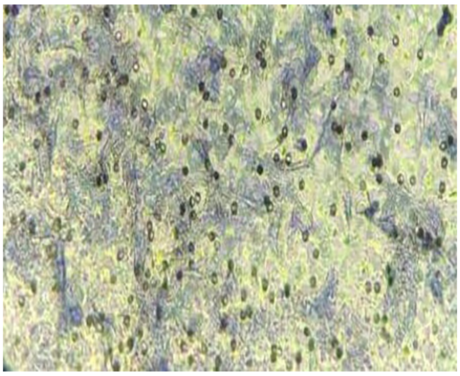
Figure S3 30-peptide control group

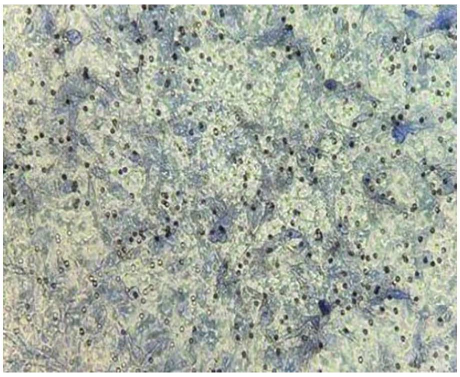
Figure S3 30-peptide 100ug/ml treating group

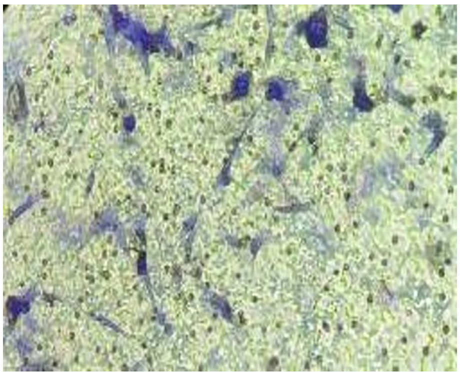
Figure S3 30-peptide 200ug/ml treating group

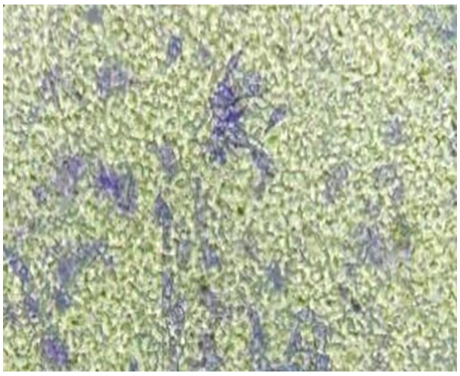
Figure S3 30-peptide 400ug/ml treating group

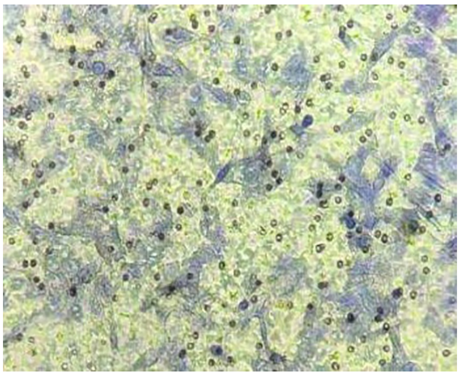

Figure S3 33-peptide control group

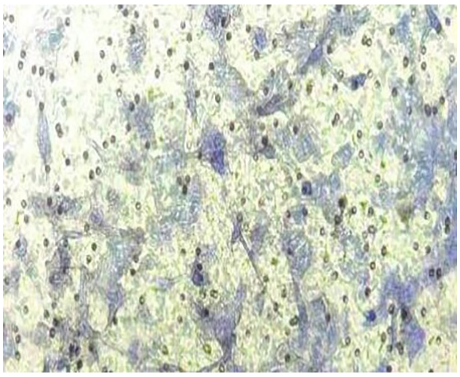

Figure S3 33-peptide 100ug/ml treating group

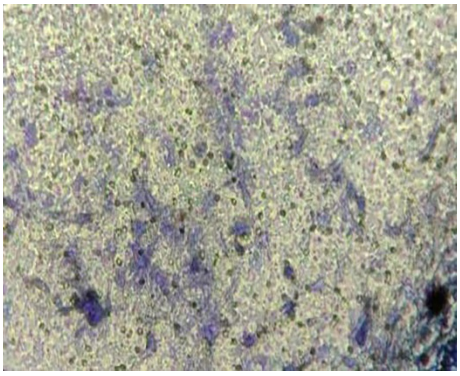

Figure S3 33-peptide 200ug/ml treating group

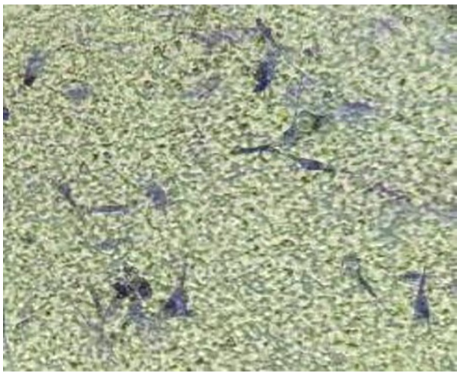

Figure S3 33-peptide 400ug/ml treating group

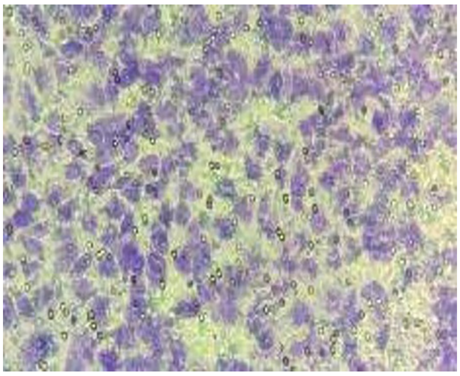

Figure S3 30-peptide control group

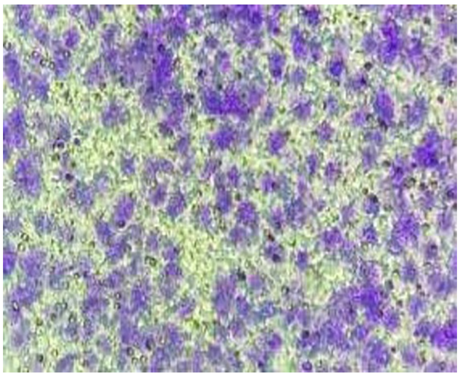

Figure S3 30-peptide 100ug/ml treating group

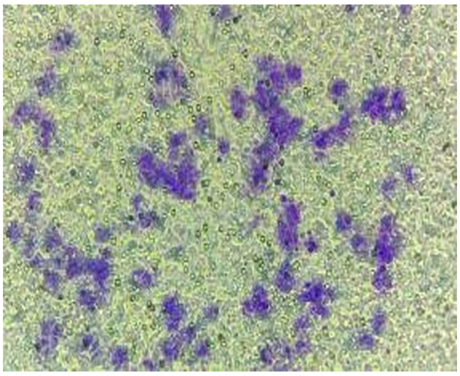

Figure S3 30-peptide 200ug/ml treating group

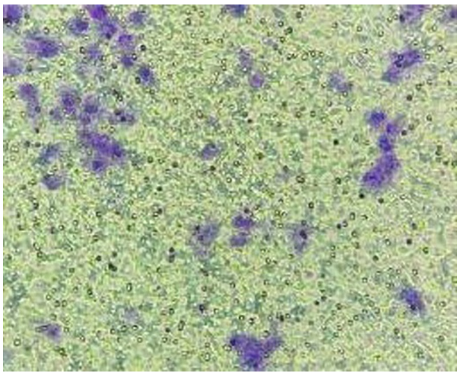

Figure S3 30-peptide 400ug/ml treating group

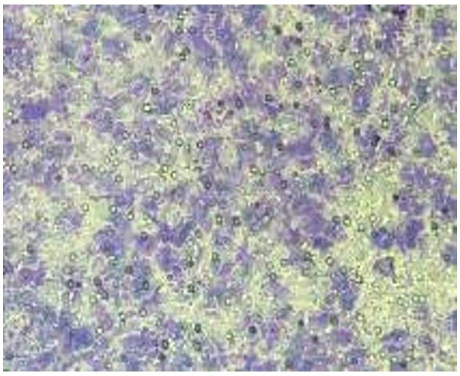
Figure S3 33-peptide control group

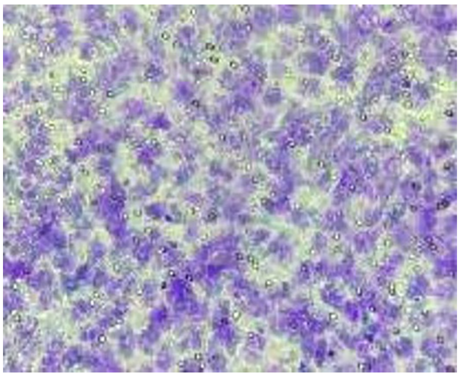
Figure S3 33-peptide 100ug/ml treating group

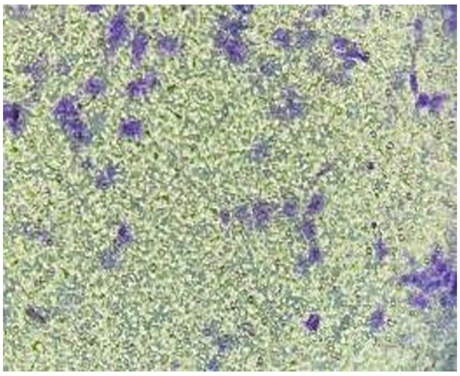
Figure S3 33-peptide 200ug/ml treating group

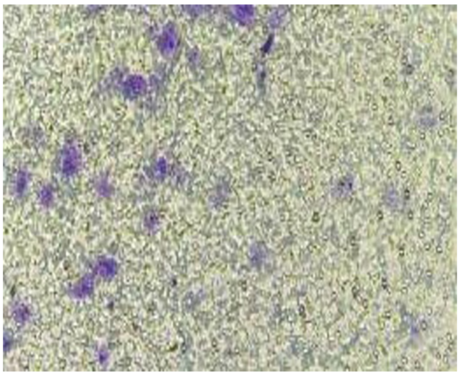
Figure S3 33-peptide 400ug/ml treating group
