# Supplementary material for: Endostatin 33 Peptide Is a Deintegrin α6β1 Agent That Exerts Antitumor Activity by Inhibiting the PI3K-Akt Signaling Pathway in Prostate Cancer
Source: J Clin Med. 2023 Feb 27;12(5):1861. doi: 10.3390/jcm12051861 (PMC10003382; doi:10.3390/jcm12051861)
Supplement: Supplementary file 1 [file jcm-12-01861-s001.zip › Supplementary Figure S1.pdf]

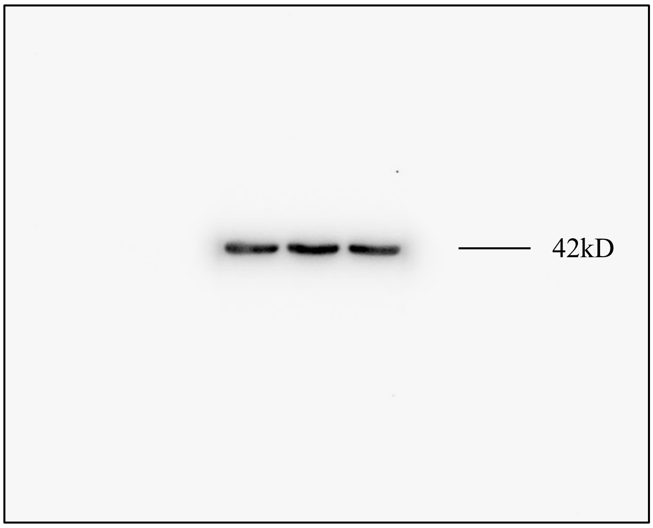

Figure 1B b-actin picture 1

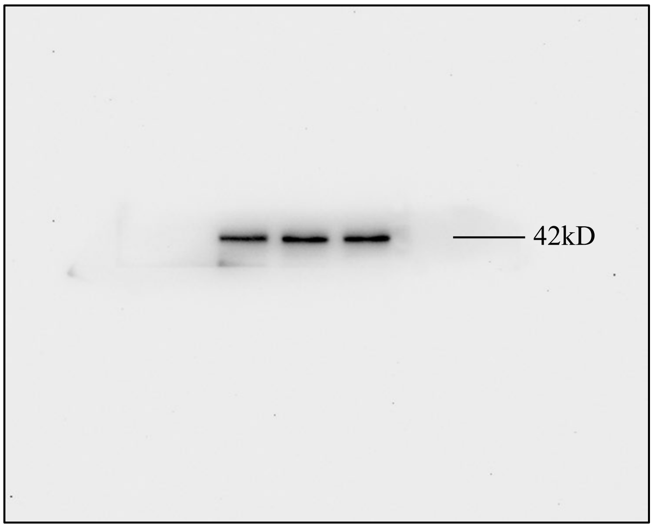

Figure 1B b-actin picture 2

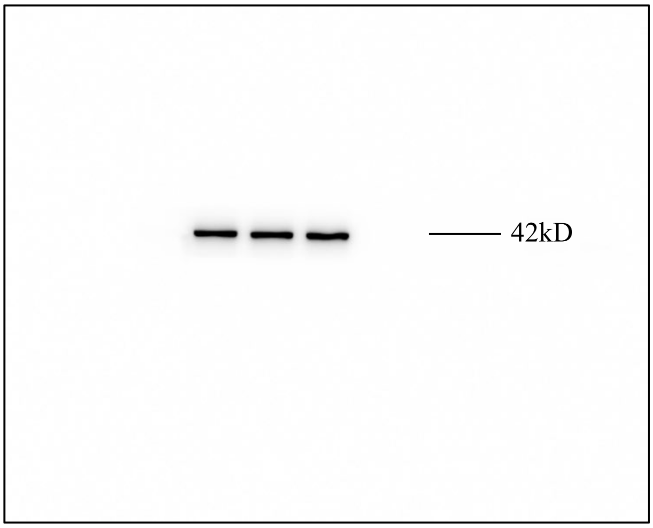

Figure 1B b-actin picture 3

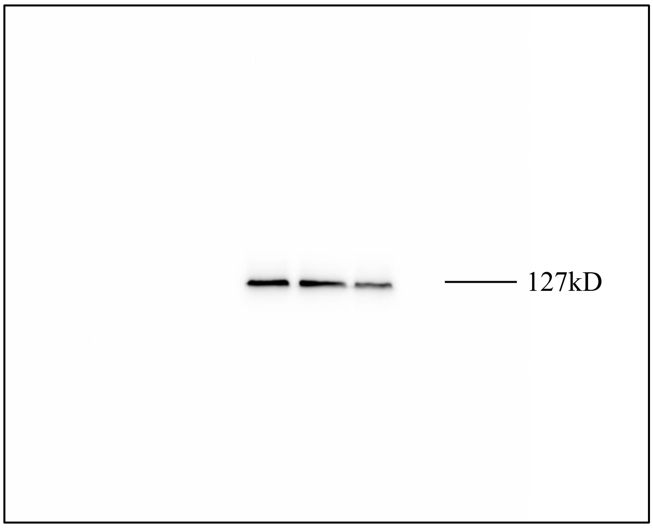

Figure 1B α6 picture 1

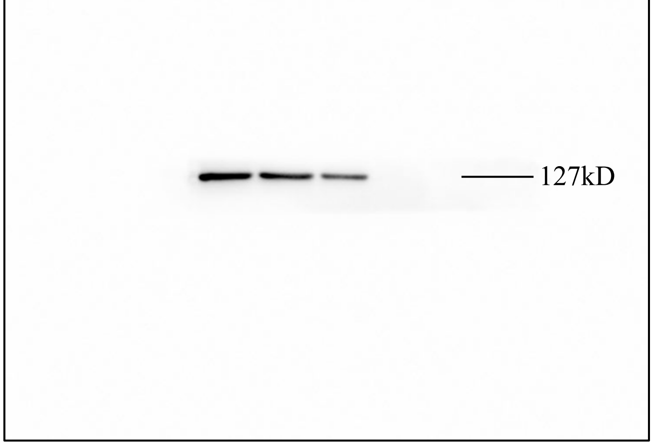

Figure 1B α6 picture 2

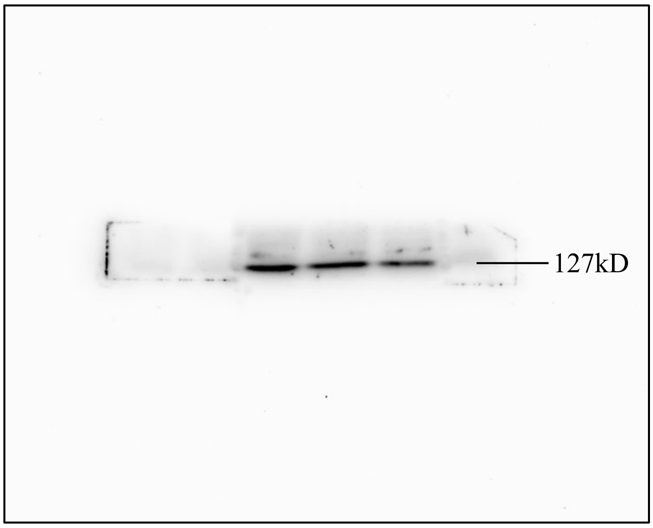

Figure 1B α6 picture 3

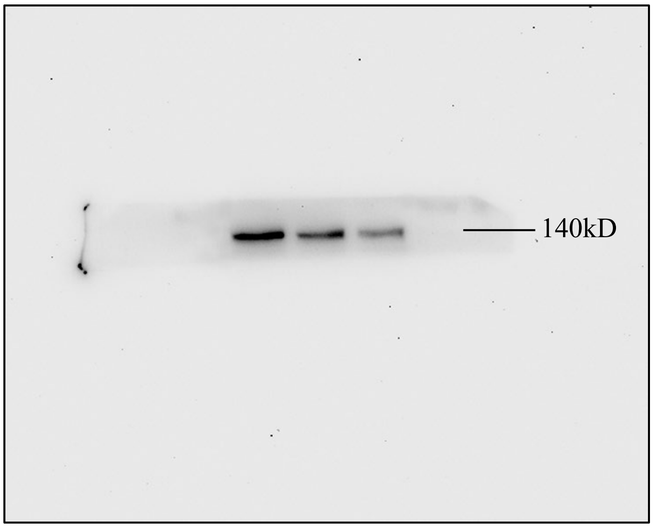

Figure 1B β1 picture 1

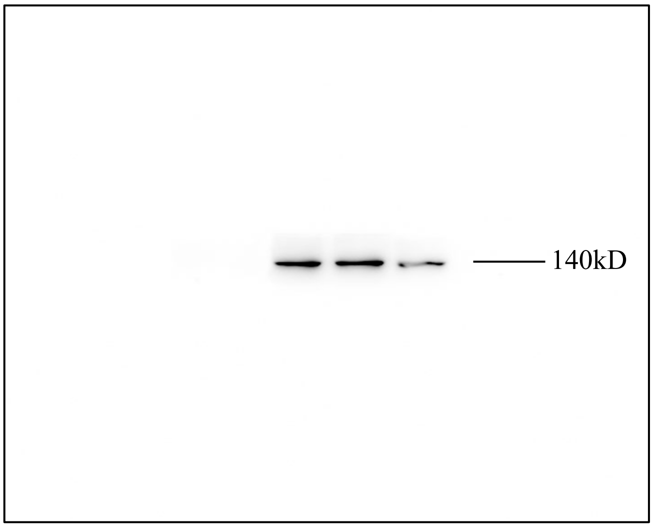

Figure 1B β1 picture 2

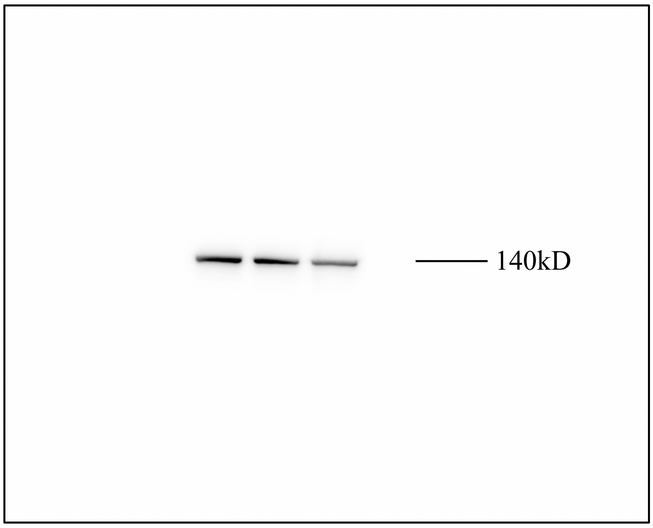

Figure 1B β1 picture 3

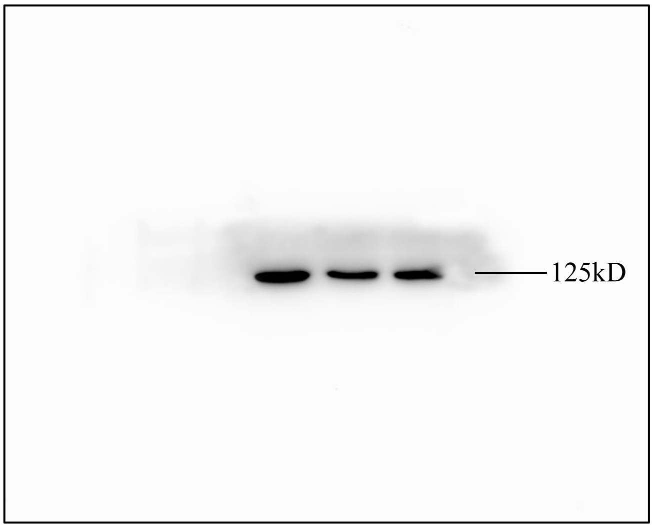

Figure 1B αv picture 1

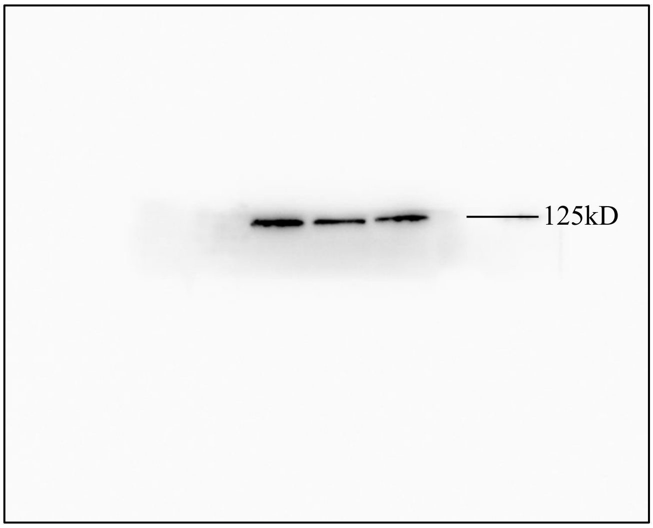

Figure 1B αv picture 2

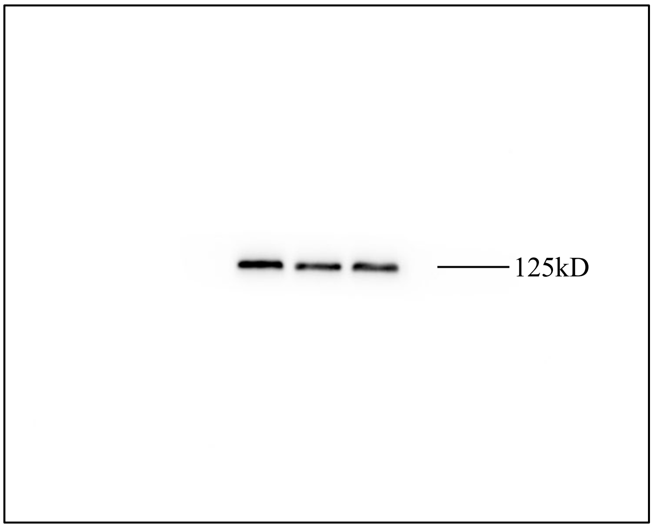

Figure 1B αv picture 3

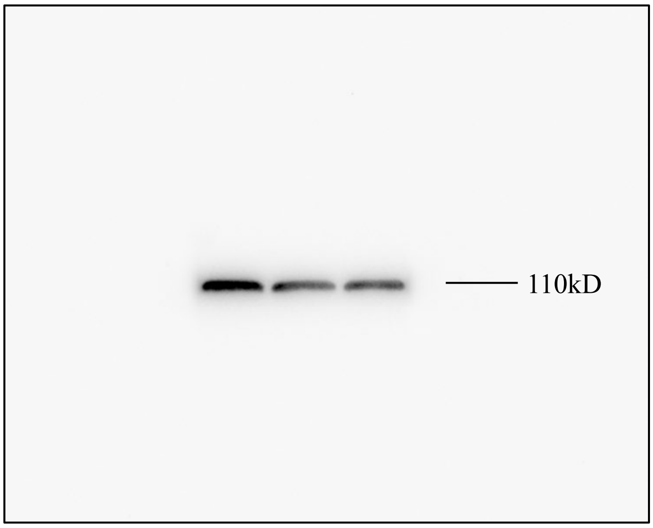

Figure 1B β3 picture 1

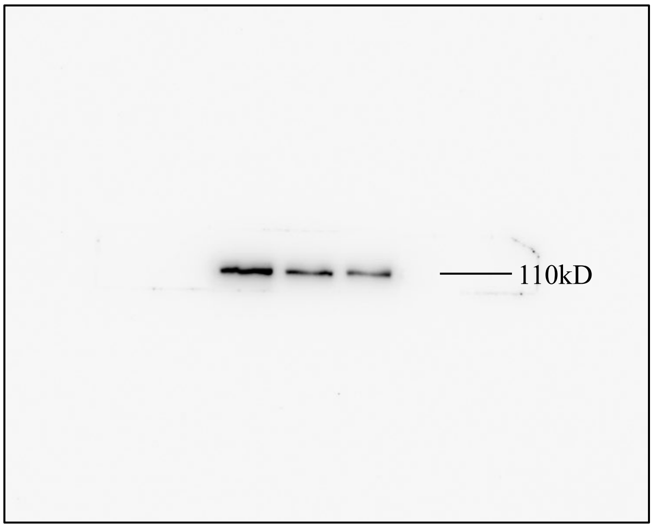

Figure 1B β3 picture 2

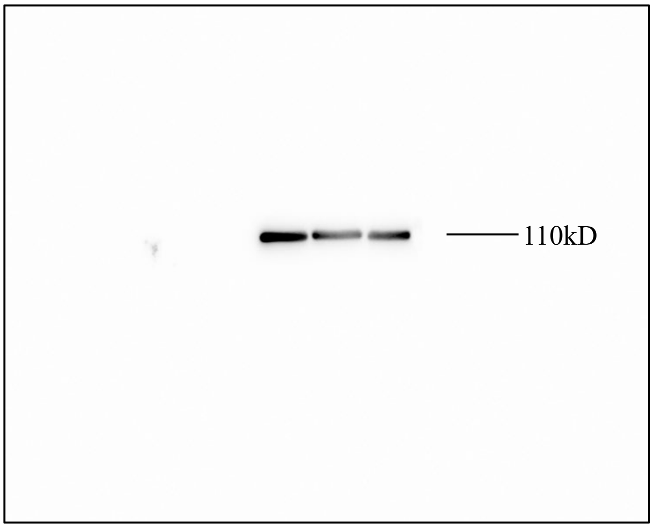

Figure 1B β3 picture 3

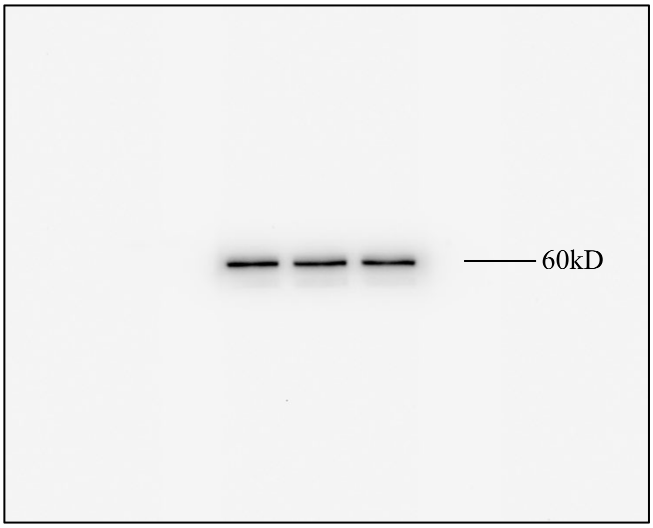

Figure 5D AKT

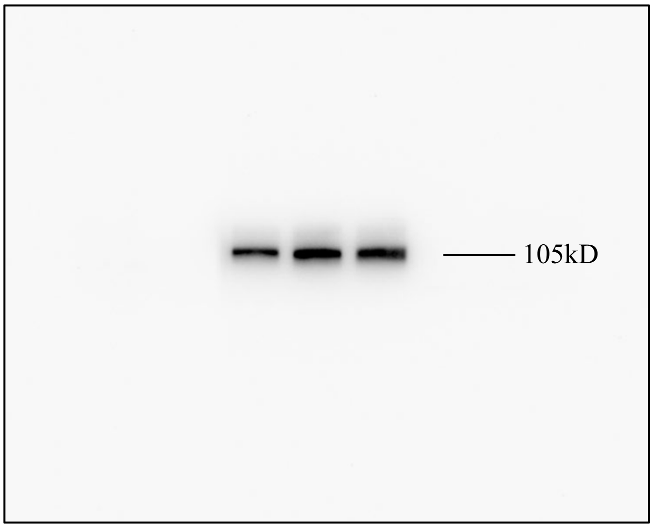

Figure 5D E-cadherin

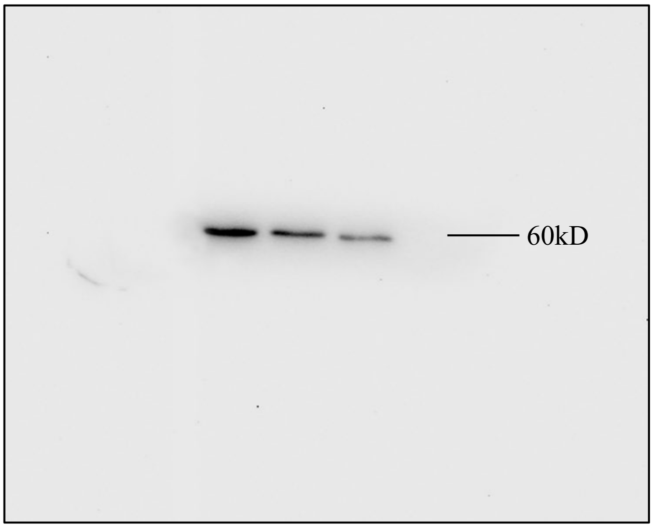

Figure 5D P-AKT

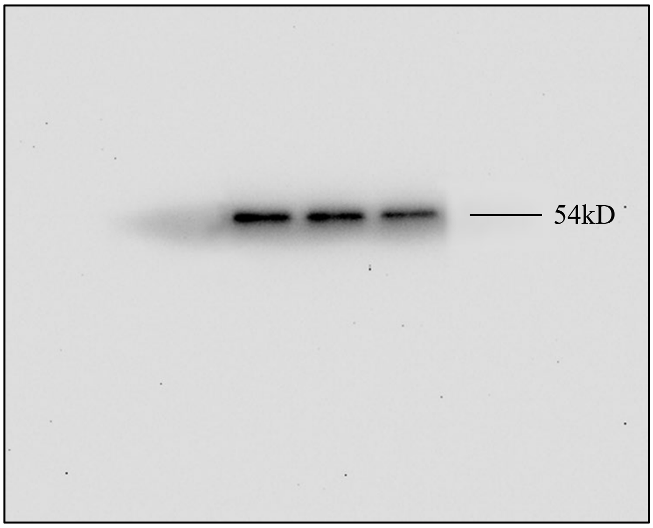

Figure 5D Vimentin

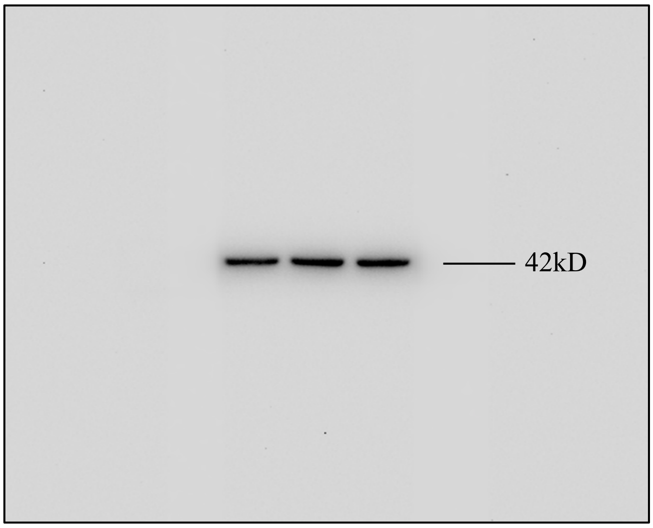

Figure 5D β-actin

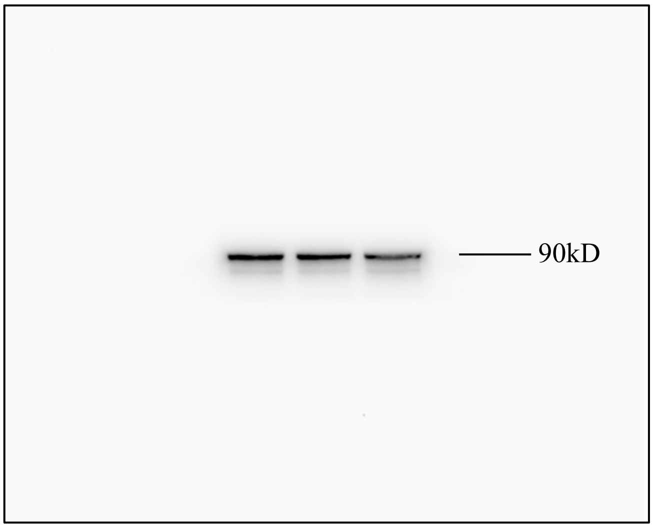

Figure 5D β-catenin

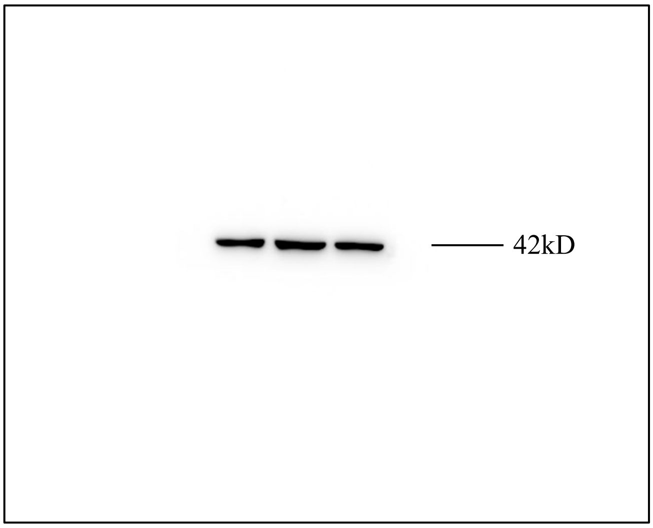

Figure 5E B-actin figure 1

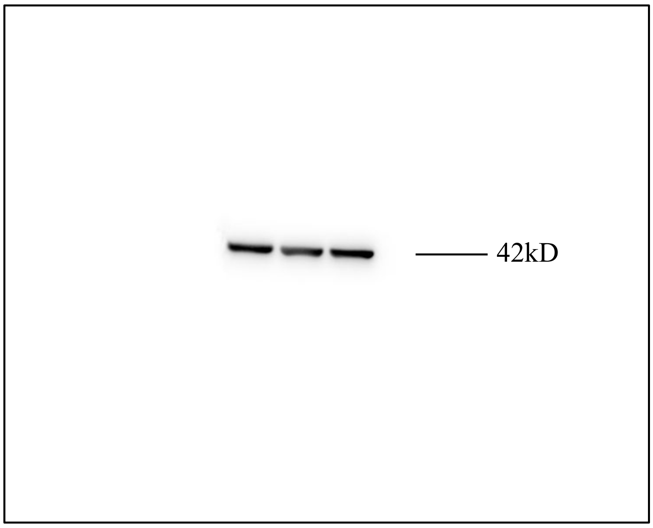

Figure 5E B-actin figure 2

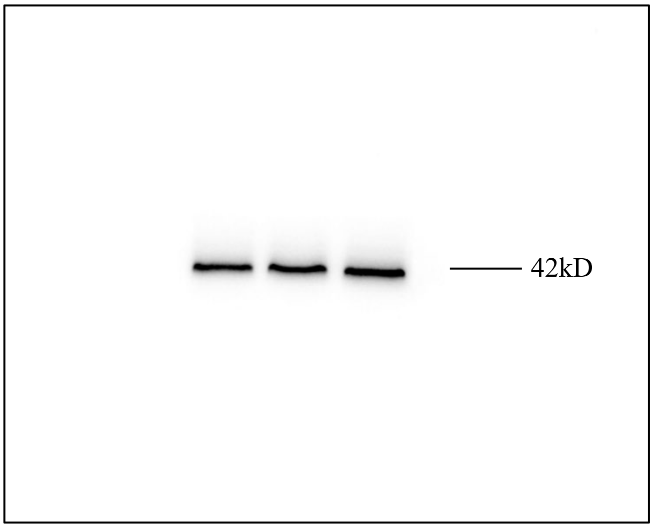

Figure 5E B-actin figure 3

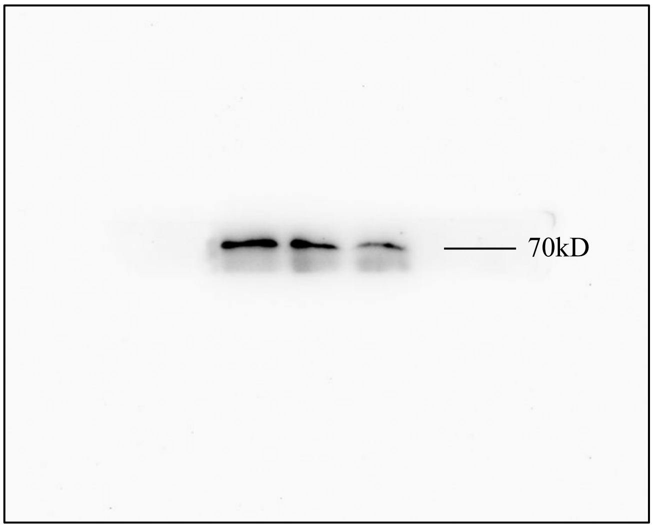

Figure 5E MMP2 picture 1

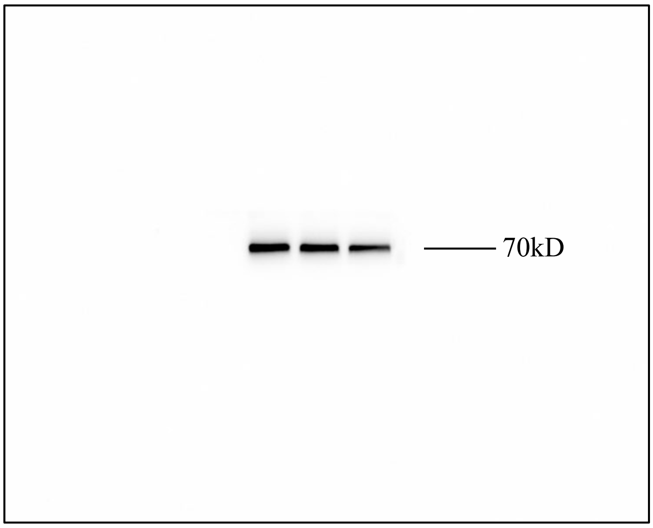

Figure 5E MMP2 picture 2

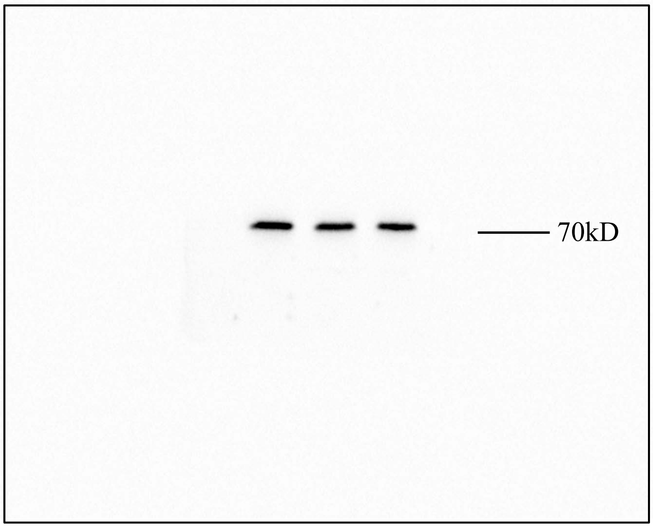

Figure 5E MMP2 picture 3

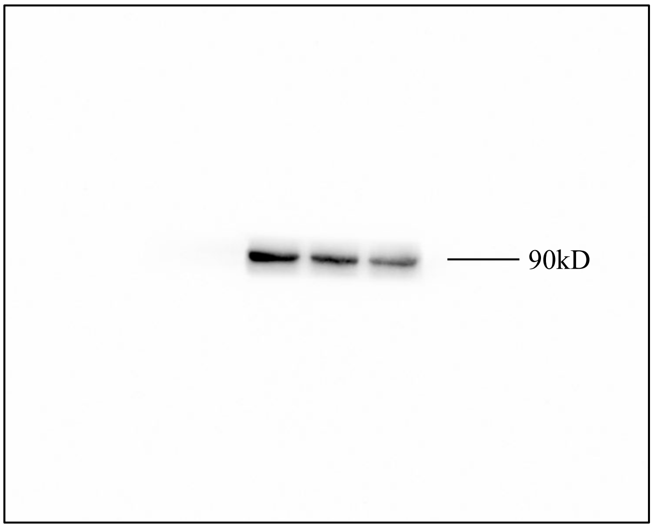

Figure 5E MMP9 picture 1

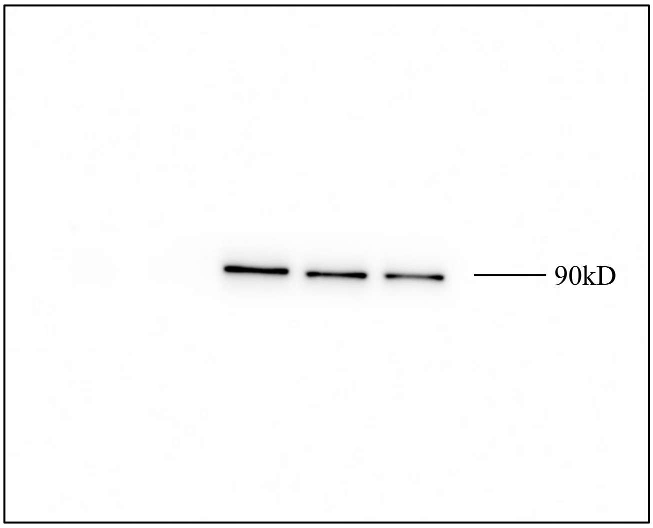

Figure 5E MMP9 picture 2

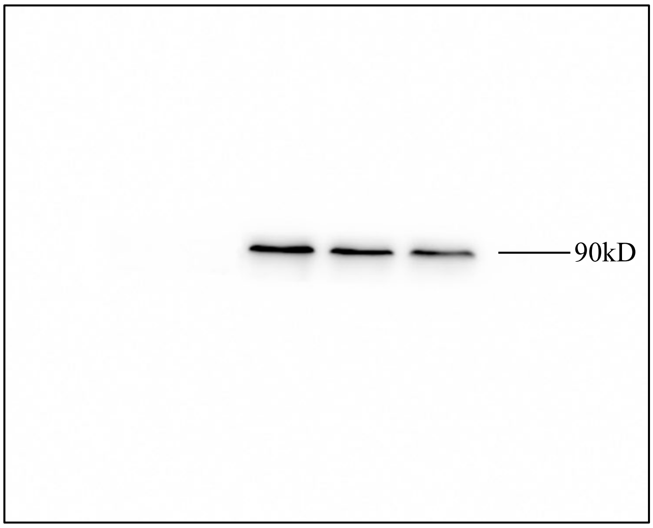

Figure 5E MMP9 picture 3

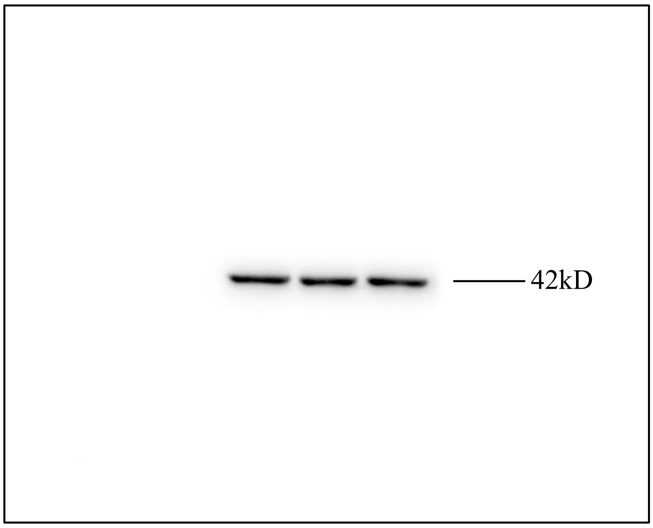

Figure 5F b-actin

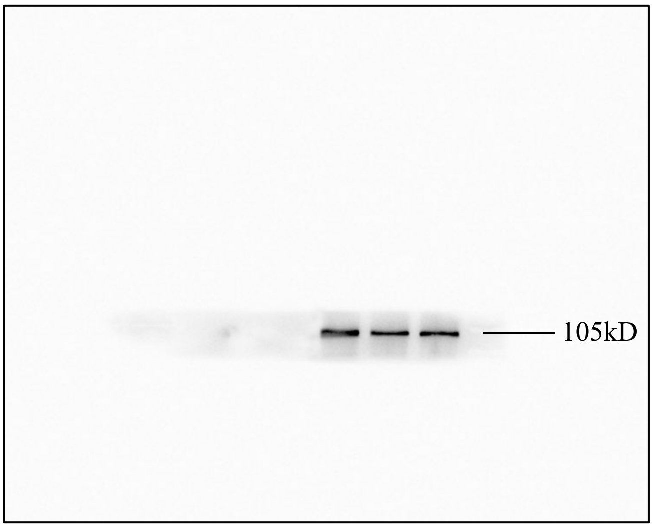

Figure 5F E-cadhenrin

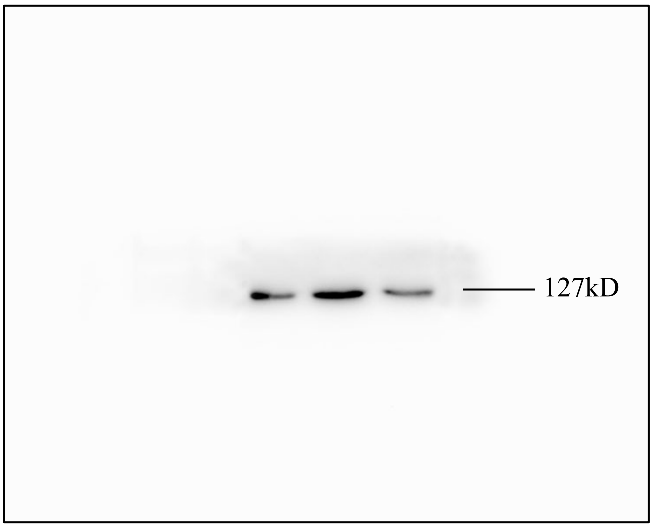

Figure 5F ITGA6

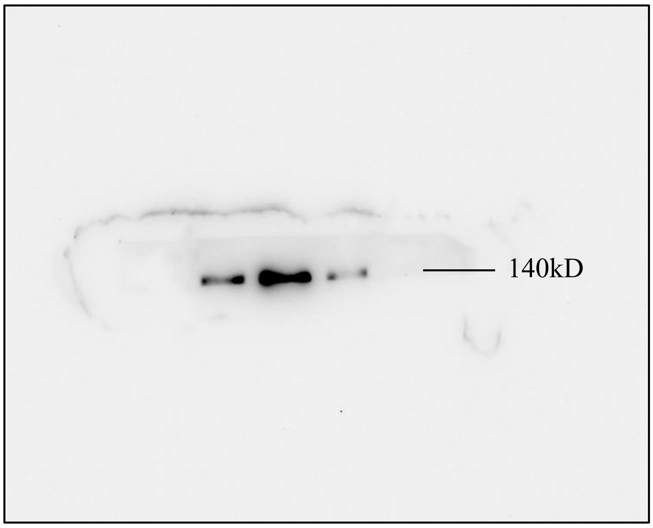

Figure 5F ITGB1

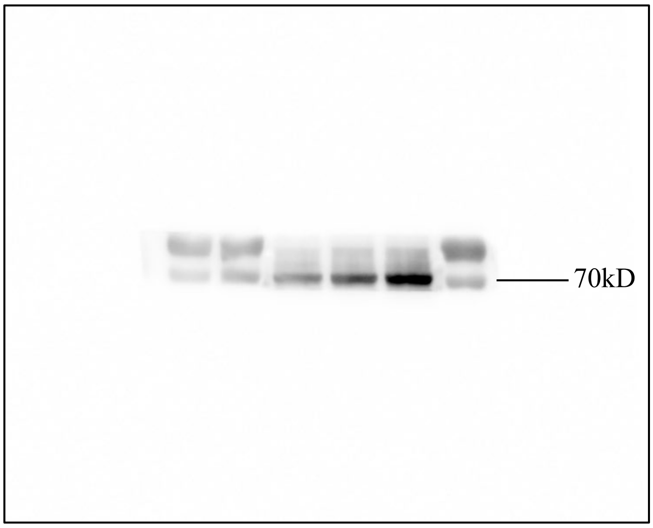

Figure 5F MMP2

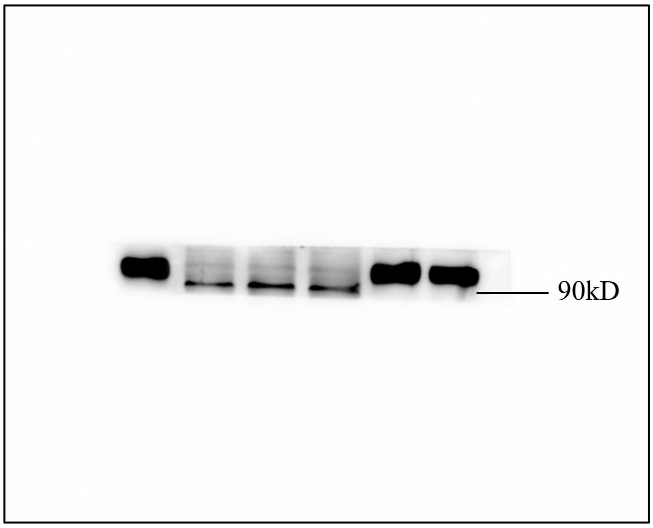

Figure 5F MMP9

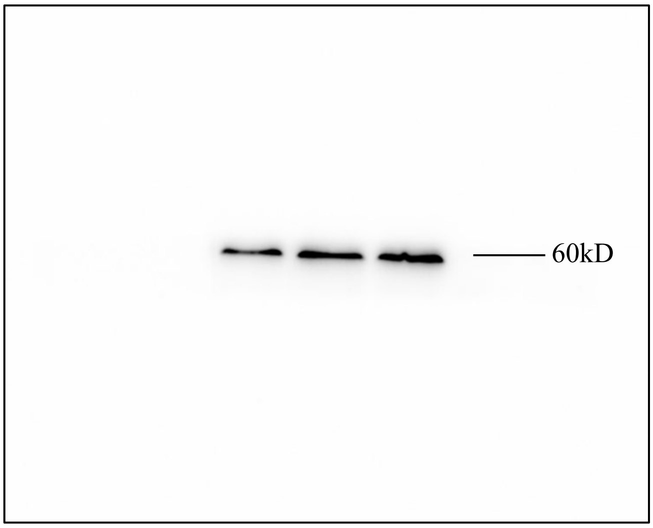

Figure 5F P-AKT

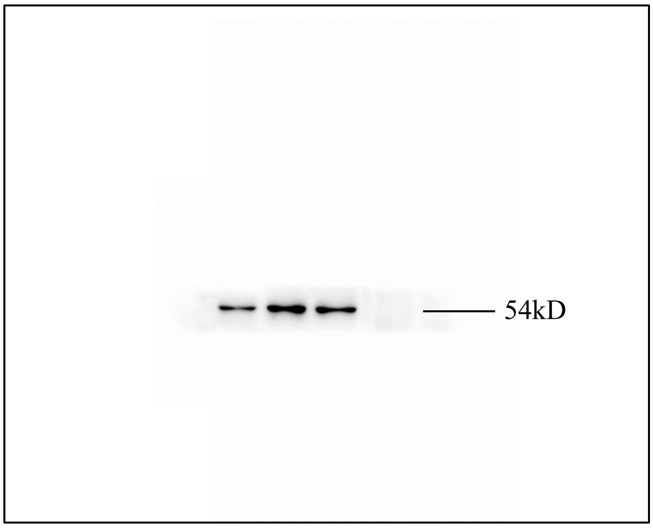

Figure 5F Vimentin

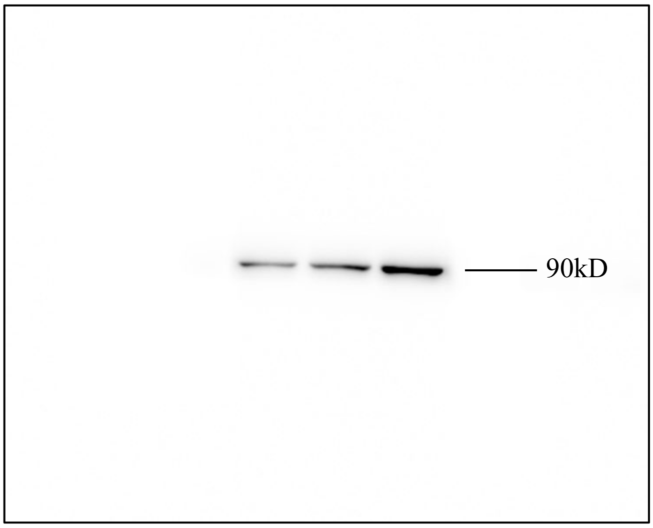

Figure 5F β-catenin
